# mRNA-specific translation regulation by a ribosome-associated ncRNA in *Haloferax volcanii*

**DOI:** 10.1038/s41598-018-30332-w

**Published:** 2018-08-21

**Authors:** Leander Wyss, Melanie Waser, Jennifer Gebetsberger, Marek Zywicki, Norbert Polacek

**Affiliations:** 1 Department of Chemistry and Biochemistry, University of Bern, Freiestrasse 3, 3012 Bern, Switzerland; 2 Graduate School for Cellular and Biomedical Sciences, University of Bern, Bern, Switzerland; 3 Department of Computational Biology, Faculty of Biology, Institute of Molecular Biology and Biotechnology, Adam Mickiewicz University, Umultowska 89, 61-614 Poznan, Poland; 4 Present Address: Institute of Organic Chemistry and Center for Molecular Biosciences (CMBI), Leopold-Franzens University, Innsbruck, Austria

## Abstract

Regulation of gene expression at the translational level allows rapid adaptation of cellular proteomes to quickly changing environmental conditions and is thus central for prokaryotic organisms. Small non-coding RNAs (sRNAs) have been reported to effectively orchestrate translation control in bacteria and archaea mainly by targeting mRNAs by partial base complementarity. Here we report an unprecedented mechanism how sRNAs are capable of modulating protein biosynthesis in the halophilic archaeon *Haloferax volcanii*. By analyzing the ribosome-associated ncRNAs (rancRNAs) under different stress conditions we identified an intergenic sRNA, termed rancRNA_s194, that is primarily expressed during exponential growth under all tested conditions. By interaction with the ribosome rancRNA_s194 inhibits peptide bond formation and protein synthesis *in vitro* but appears to target a specific mRNA *in vivo*. The respective knock-out strain shows a reduced lag phase in media containing xylose as sole carbon source and outcompetes the wildtype cells under these conditions. Mass spectrometry, polysome profiling and mRNA binding competition experiments suggest that rancRNA_s194 prevents the *cstA* mRNA from being efficiently translated by *H*. *volcanii* ribosomes. These findings enlarge the regulatory repertoire of archaeal sRNAs in modulating post-transcriptional gene expression.

## Introduction

Non-protein-coding RNAs (ncRNAs) have been identified in all three domains of life and have been shown to be important for regulation of many different biological processes (reviewed in refs^1,2^). In contrast to the many reports about the functional and mechanistic roles of ncRNAs in eukaryotes or bacteria, much less is known about small ncRNAs (sRNAs) in archaea. Nevertheless, recent studies have identified potential *cis* and *trans* acting sRNAs in high throughput RNA sequencing approaches in a number of archaeal organisms including *Haloferax volcanii* (reviewed in^3^). *H*. *volcanii* is a halophilic archaeal model organism belonging to the phylum Euryarchaeota. Its genome consists of a main chromosome (2.848 Mb), three smaller chromosomes (85.1 kb, 636 kb, 938 kb) and one plasmid (6.35 kb)^4^. In a recent attempt to identify transcriptional start sites (TSSs) with differential RNA-sequencing in the halophilic archaeon *H*. *volcanii* under optimal growth conditions, 2,900 TSSs identified belonged to putative sRNA genes^5^. This is a rather unexpectedly high number considering that only 4,040 proteins are annotated in the *H*. *volcanii* genome, but underlining the still mostly hidden biological potential of ncRNA transcripts. Most of these potential sRNAs identified were oriented in the sense or antisense orientation relative to the respective mRNAs and for some of these sRNAs correlative studies have shown altered sRNA expression under certain stress conditions^6^. Nevertheless conclusive experimental validation showing that sense or antisense sRNAs in archaea are functional for mRNA translation regulation are sparse. In the recent RNA sequencing study in *H*. *volcanii*^5^ also 400 intergenic sRNAs were found. It has been demonstrated that in archaea some intergenic sRNA genes are differently expressed in response to growth in varying environmental stress conditions^6–8^. Phenotypic characterization of sRNA deletion mutants confirmed the importance of some of the intergenic sRNAs in *H*. *volcanii* biology^8,9^, but no direct cellular targets of these intergenic sRNAs have been experimentally examined so far. In other archaeal organisms also only a small number of intergenic sRNA targets have been studied; e.g. the sRNA 154 in *Methanosarcina mazei*. This sRNA is exclusively expressed during nitrogen starvation conditions and was found to stabilize some mRNAs involved in stress response while inhibiting translation of others^10^.

The above mentioned example of sRNA translation regulation in archaea, as well as all the intensively studied other ncRNA classes like miRNAs, siRNAs or antisense RNAs, have one mechanistic feature in common: they all target the mRNA via base complementarity in order to modulate protein biosynthesis. In recent years although, a novel class of ncRNAs involved in translation regulation was found in various organisms, the so called ribosome associated non-coding RNAs (rancRNAs) (reviewed in^11^). RancRNAs directly target the ribosome, the central enzyme of protein biosynthesis, and were shown to have stress specific expression patterns or different association behavior with the ribosome^12,13^. These rancRNAs were capable of inhibiting translation of proteins on a global level, e.g. by competing with mRNA binding to the ribosome^14^. These rancRNAs were not encoded as separate genes but they represent processing products deriving from longer precursor transcripts of known function, namely mRNAs^13^ or tRNAs^12,14^. In this study we revealed intergenic rancRNA candidates in *H*. *volcanii* that most likely represent genuine ncRNA genes. In particular we revealed rancRNA_s194 as translation regulator by associating with polysomes during exponential growth thereby excluding a distinct mRNA from being translated. This is an unprecedented function of a rancRNA and enlarges the regulatory repertoire of this emerging class of regulatory molecules.

## Results

### The non-coding RNA interactome of *H*. *volcanii* ribosomes

**Figure 1:**
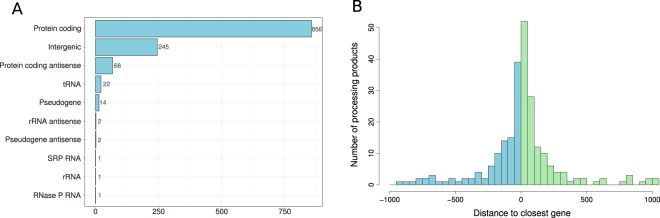
Summary of the annotation of predicted RNA processing products from the *H*. *volcanii* rancRNA library. (**A**) Display of the numbers of processing product annotated to different genomic features. (**B**) Distance from novel intergenic rancRNA candidates to the closest gene (in nucleotides). Values below zero (blue) reflect the genes located upstream from the rancRNA candidate locus, values above zero (green) show genes downstream from rancRNA candidate.

In order to identify ribosome -associated noncoding RNAs (rancRNAs) in *H*. *volcanii*, a cDNA library was prepared from small RNAs (20–500 nt) which co-purify with ribosomal particles isolated from *H*. *volcanii* cells grown under different environmental stress conditions^12^. Deep sequencing yielded 73.5 million raw reads which were then further analyzed with the automated computational pipeline, named APART^15^. After the bioinformatics clean-up we ended up with 26.2 million reads which were then mapped to the reference genome (*Haloferax volcanii* DS2 Mar 30 2010) as a guide for assembly and annotation of the cDNA sequences. This procedure predicted 1,212 rancRNA candidates in *H*. *volcanii*. The obtained sequence reads have been deposited in ENA (Acc. No PRJEB25902). The vast majority of putative rancRNA candidates appear to be processed from longer functional precursor transcripts such as mRNAs or tRNAs, but also a substantial group originates from intergenic regions of the genome (Fig. 1A). Only a minor fraction corresponds to antisense transcripts.

### Several intergenic rancRNA candidates identified in the library are expressed in *H*. *volcanii* cells

In this study we focused on the functional characterization of intergenic rancRNA candidates, and thus representing putative novel ncRNA genes, which had high read numbers in the cDNA library. High sequence read numbers are indicative for active gene expression and high transcript abundance in *H*. *volcanii* cells. Furthermore, we also only considered rancRNA candidates which had read stacks with defined ends on both sides. This is symptomatic for individually transcribed genes with defined transcription start and termination sites or for stable and precise post-transcriptional processing from longer RNA precursors. Here we report on three RNAs which were among the ten most abundant intergenic candidates identified in the rancRNA library of *H*. *volcanii*.

**Figure 2:**
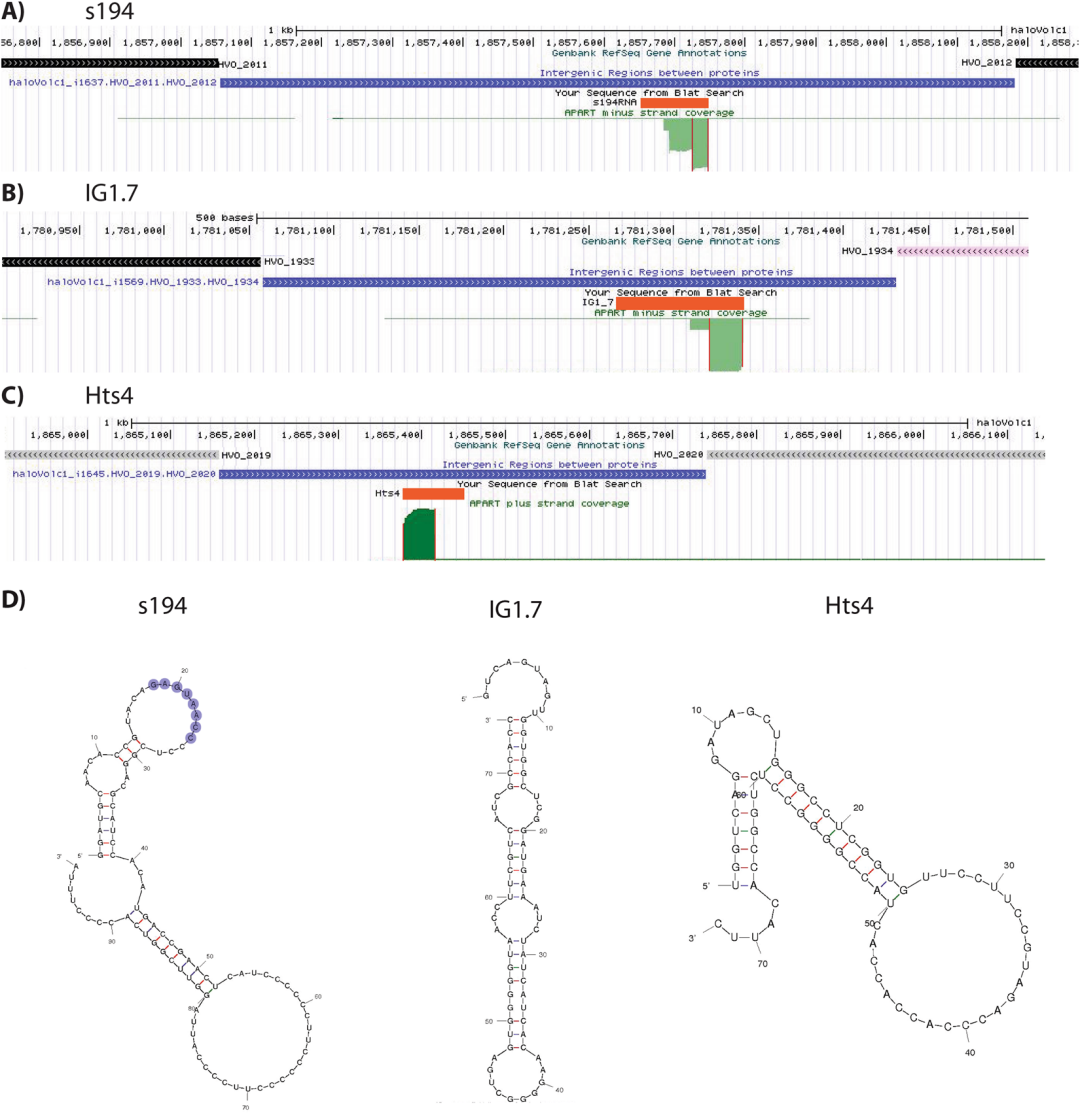
Genomic location and secondary structure prediction of selected intergenic rancRNA candidates. (**A**,**B**,**C**) Genome browser views of the locations of the rancRNA candidates s194, IG1.7 and Hts4 in the *H*. *volcanii* genome. In green the coverage of the sequencing results are shown. Thin vertical red lines indicate the borders of the predicted processing products by the APART pipeline. The orange bars show the respective lengths of rancRNA candidates as determined by 3′-RACE. Blue boxes indicate intergenic regions of the *H*. *volcanii* genome whereas up- and downstream ORFs are indicated by black, rose and grey boxes, respectively, with arrows indicating the orientation of the genes. (**D**) Secondary structure predictions by Mfold of rancRNA candidates s194, IG1.7 and Hts4 are shown. The residues highlighted in purple in s194 are complementary to the putative mRNA target cstA.

**Figure 3:**
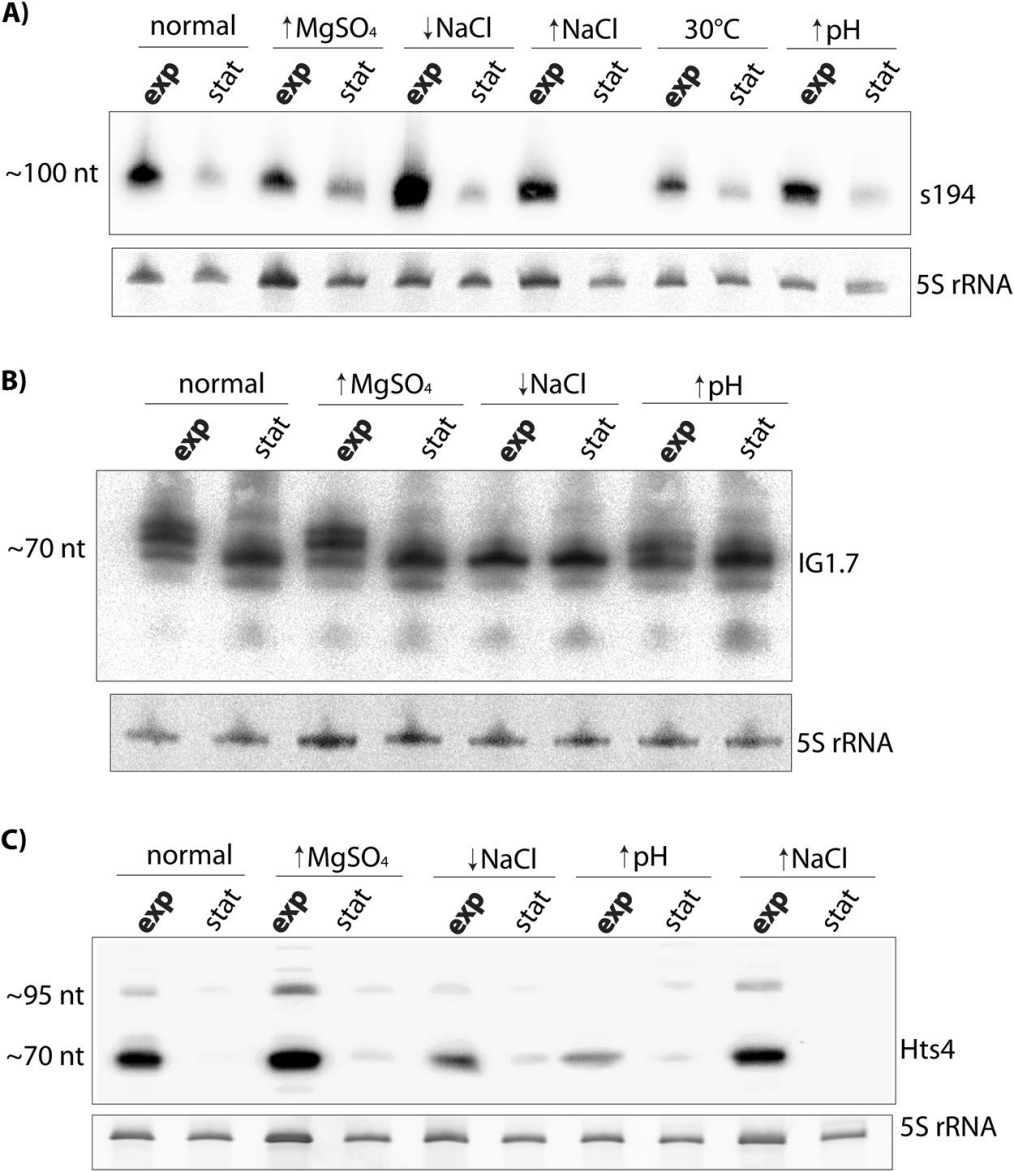
Expression of rancRNA candidates in *H*. *volcanii*. Northern blot analyses of rancRNA candidates s194 (**A**), IG1.7 (**B**) and Hts4 (**C**) on total RNA isolated from *H*. *volcanii* wildtype cells grown to either exponential (exp) or stationary (stat) phase. *H*. *volcanii* cells were grown at 42 °C or 30 °C in normal or in stress medium (↑MgSO_4_, high magnesium concentration; ↑or ↓NaCl, high or low sodium concentration; ↑pH, alkaline stress). 5 S rRNA serves as loading control. Full-length blots are presented in Supplementary Fig. 9.

The first rancRNA candidate was predicted by the APART pipeline as a 23 nucleotides long molecule which derives from the minus strand of the main chromosome of the *H*. *volcanii* genome (Fig. 2A). Based on the read numbers, this RNA was by far the most abundant intergenic rancRNA candidate. When testing the expression of this RNA with northern blot analysis using total RNA from *H*. *volcanii* cells we detected mainly one intensive signal with a length of about 100 nucleotides (Fig. 3A). This RNA was also found in a library prepared from total RNA in *H*. *volcanii* in a previous study and was dubbed s194 (ref.^8^). Looking at the sequencing reads (Fig. 2A), additional longer but less abundant reads were apparent. All reads possess several cytidines in the 3′ end (4–6 nt C stretches) that probably served as internal annealing sites for the oligo(dG)-RT-primer used for cDNA construction. Therefore during cDNA preparation mainly the shortest construct was amplified and subsequently sequenced. With help of 3′ rapid amplification of cDNA ends (3′-RACE) we determined the length of the s194 RNA to be 97 nucleotides (orange bar in Fig. 2A) which is in accordance with the length of the RNA detected in northern blot analysis. The sequence and the predicted secondary structure by mfold^16^ of s194RNA are shown in Fig. 2D. The s194 RNA was primarily detectable during the exponential growth phase and was significantly less abundant during stationary phase (Fig. 3A). This expression pattern was similar regardless of the growth conditions tested.

The next intergenic rancRNA candidate was predicted to be 19 nucleotides long and derives from the minus strand of the main chromosome of the *H*. *volcanii* genome (Fig. 2B). Based on its location on the chromosome this RNA was named IG1.7. Also in this case the predicted stable processing product has a stretch of five cytidines at the predicted 3′ end and could therefore enable internal alignment of the oligo(dG)-RT-primer. To determine the full-length IG1.7 RNA sequence, we performed 3′-RACE and determined the RNA to be 74 nucleotides (orange bar in Fig. 2B) with the predicted secondary structure being dominated by stems and only a few unpaired residues (Fig. 2D). When testing the expression of the IG1.7 RNA with northern blot analysis two or under some growth conditions four bands were detected (Fig. 3B). The strongest signal came from a band with a length of around 70 nucleotides which is in accordance with the 3′-RACE results. The intergenic RNA IG1.7 is equally expressed under all growth conditions tested and was more or less equally abundant in the exponential and stationary growth phase. It became clear that the length of this RNA seems to vary slightly between different growth conditions. IG1.7 overlaps with a predicted 30 residue long RNA (named htsf242) that was found by deep sequencing of a cDNA library of total *H*. *volcanii* RNA^9^. We never detected a signal on the northern blot at this length and so far no other published experimental data confirmed the existence of this 30 nucleotide long htsf242 RNA.

The third investigated intergenic rancRNA candidate was predicted with a length of 36 nucleotides and derives from the plus strand of the main chromosome of the *H*. *volcanii* genome (Fig. 2C). However, 3′-RACE revealed the actual length of this RNA to be 72 residues long (Fig. 2C,D). The RNA expression was tested with northern blot analysis on total RNA isolated from *H*. *volcanii* cells grown under different growth conditions. In accordance with the 3′-RACE results, a band with an estimated length of about 70 nucleotides became evident (Fig. 3C). An additional and clearly less abundant band at around 95 residues was detectable which might represent a precursor of this rancRNA candidate. The RNA was strongly expressed during exponential growth but was essentially absent in the stationary phase (Fig. 3C). Overall expression of this RNA seems to be inhibited in low NaCl containing media and during alkaline stress. This RNA was also found previously in a study aimed to identify the complete small RNome of *H*. *volcanii*^7^ and therein it was named Hts4 RNA. In this study, multiple bands were visible in northern analysis at the length of 65, 75 and 110 residues whereas in our hands solely one major band around 70 nucleotides and a faint band at 95 nucleotides were observed (Fig. 3C).

### Association of rancRNA candidates with *H*. *volcanii* ribosomes

**Figure 4:**
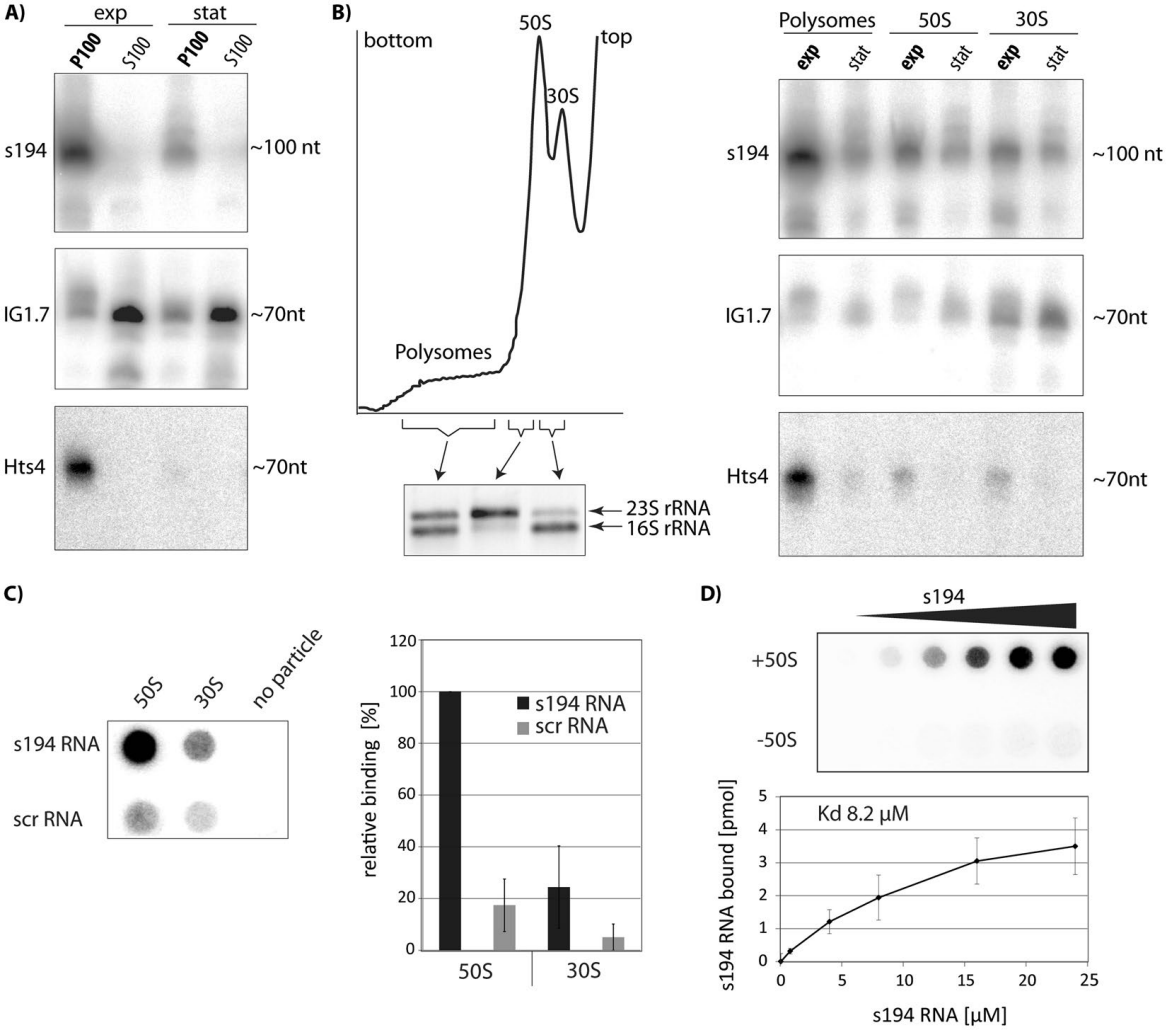
s194 and Hts4 RNAs interact with ribosomes. (**A**) Northern blot analysis of intergenic rancRNA candidates on RNA isolated from crude ribosome pellets (P100) or the corresponding supernatants (S100) of *H*. *volcanii* cells grown in normal medium to either exponential (exp) or stationary (stat) phase. (**B**) On the left a representative polysome profile of *H*. *volcanii* is shown. Fractions containing polysomes, 50S, or 30S subunits were analyzed on an agarose gel to confirm the rRNA identities. On the right northern blot signals for different rancRNA candidates in different gradient fractions are shown. *H*. *volcanii* cells were either grown to exponential (exp) or stationary (stat) phase. Full-length blots are presented in Supplementary Fig. 9. (**C**) *In vitro* filter binding studies of [^32^P]-radiolabeled *in vitro* transcribed s194 RNA on purified ribosomal subunits (left panel, representative phosphorimager screen). As specificity control, an *in vitro* transcribed radiolabeled RNA with same length, but randomized nucleotide sequence (scr) was used. Right panel shows the mean and standard deviations of four experiments whereas binding of s194 RNA to 50S ribosomal subunit was set to 100%. (**D**) Increasing amounts of [^32^P]-radiolabeled *in vitro* transcribed s194 RNA were added to 5 pmol purified *H*. *volcanii* 50S ribosomal subunits and binding efficiency was monitored by dot blot filter binding. The signals obtained in the absence of any ribosomal subunits (−50S) served as background and were subtracted from the corresponding samples containing 50S subunits (+50S). Lower panel shows the mean and standard deviation of three independent binding experiments.

To test ribosome-association of the three rancRNA candidates, ribosomes were pelleted from *H*. *volcanii* cell lysates by ultracentrifugation at 100,000 × *g*. Northern blot analysis revealed that the s194 RNA is almost exclusively found in the crude ribosomal pellet fraction (P100) and was basically absent in the corresponding supernatant (S100) (Fig. 4A). To investigate to which ribosomal particle the s194 RNA is associated *in vivo*, polysome profiling followed by northern blot analysis was performed. RancRNA candidate s194 was mainly found in the polysome fractions of exponentially growing cells (Fig. 4B) and therefore seems to bind to actively translating ribosomes. s194 RNA was also found in the fractions with 50S and 30S ribosomal subunits from exponentially growing cells but to a much lesser extent. As seen previously^12^, 70S ribosomes were missing in the polysome gradients (Fig. 4B) most likely due to their reduced stability in the high salt sucrose gradient conditions. To investigate if the s194 RNA can bind to isolated ribosomal subunits, *in vitro* transcribed s194 RNA was radioactively labeled and incubated with density gradient-purified 50S and 30S ribosomal subunits isolated from *H*. *volcanii* cells. To assure a completely unoccupied s194 RNA binding site, ribosomal subunits were isolated from a *H*. *volcanii* s194 knock-out strain^17^ lacking expression of this ncRNA (Supplementary Fig. 1). Ribosomal subunits and added s194 RNA were then incubated and the formed complexes analyzed via filter binding. In this setup, s194 RNA primarily bound to the large ribosomal subunit, whereas the scrambled control RNA showed only weak association (Fig. 4C). These findings highlight the specificity of the s194 RNA/ 50S interaction. Binding saturation experiments revealed a single binding site on the 50S ribosomal subunit (Fig. 4D). Collectively these data demonstrate that s194 is a genuine ribosome-associated ncRNA and we will therefore refer to this molecule as rancRNA_s194.

Association of the rancRNA candidates IG1.7 and Hts4 with the *H*. *volcanii* ribosome was assessed in an analogous manner. In contrast to rancRNA_s194, only a minor fraction of IG1.7 transcripts were associated with ribosomes *in vivo* (Fig. 4A,B). To test if the IG1.7 RNA plays a physiological role in *H*. *volcanii*, we knocked out the corresponding genomic locus and performed growth curves under different environmental conditions. While in most of the tested conditions (high Mg^2+^, alkaline pH, heat shock, high salt, various carbon sources) the knock-out strain had no significant deficiencies, incubation in low NaCl medium revealed a slightly inhibited growth of the knock-out strain relative to the wildtype cells (Supplementary Fig. 2A). To further assess the biological fitness of the IG1.7 knock-out strain, growth competition experiments were performed. In low NaCl media condition the knock-out strain was rapidly outcompeted by the wildtype *H*. *volcanii* (Supplementary Fig. 2B). On the other hand, under normal growth conditions cells lacking the IG1.7 RNA showed no fitness deficits. These results demonstrate that the IG1.7 ncRNA is important for proper growth of *H*. *volcanii* under low salt stress conditions, but most likely does not modulate translation by directly binding to the ribosome. On the contrary, the rancRNA candidate Hts4 showed a strong association with ribosomal particles and seems to mainly interact with polysomes in exponentially growing *H*. *volcanii* cells (Fig. 4A,B). *In vitro* translation reactions in the presence of Hts4 RNA showed inhibition of protein synthesis, but peptide bond formation data were ambiguous (Supplementary Fig. 3). A knock-out strain of rancRNA candidate Hts4 has been generated previously, which however did not show a strong growth phenotype under various stress conditions as compared to the parental cells^7^. Based on the fact that IG1.7 and Hts4 do not associate with ribosomes or do not reproducibly affect model reactions of protein biosynthesis, respectively, we subsequently focused our investigations on rancRNA_s194.

### rancRNA_s194 RNA inhibits *in vitro* translation

**Figure 5:**
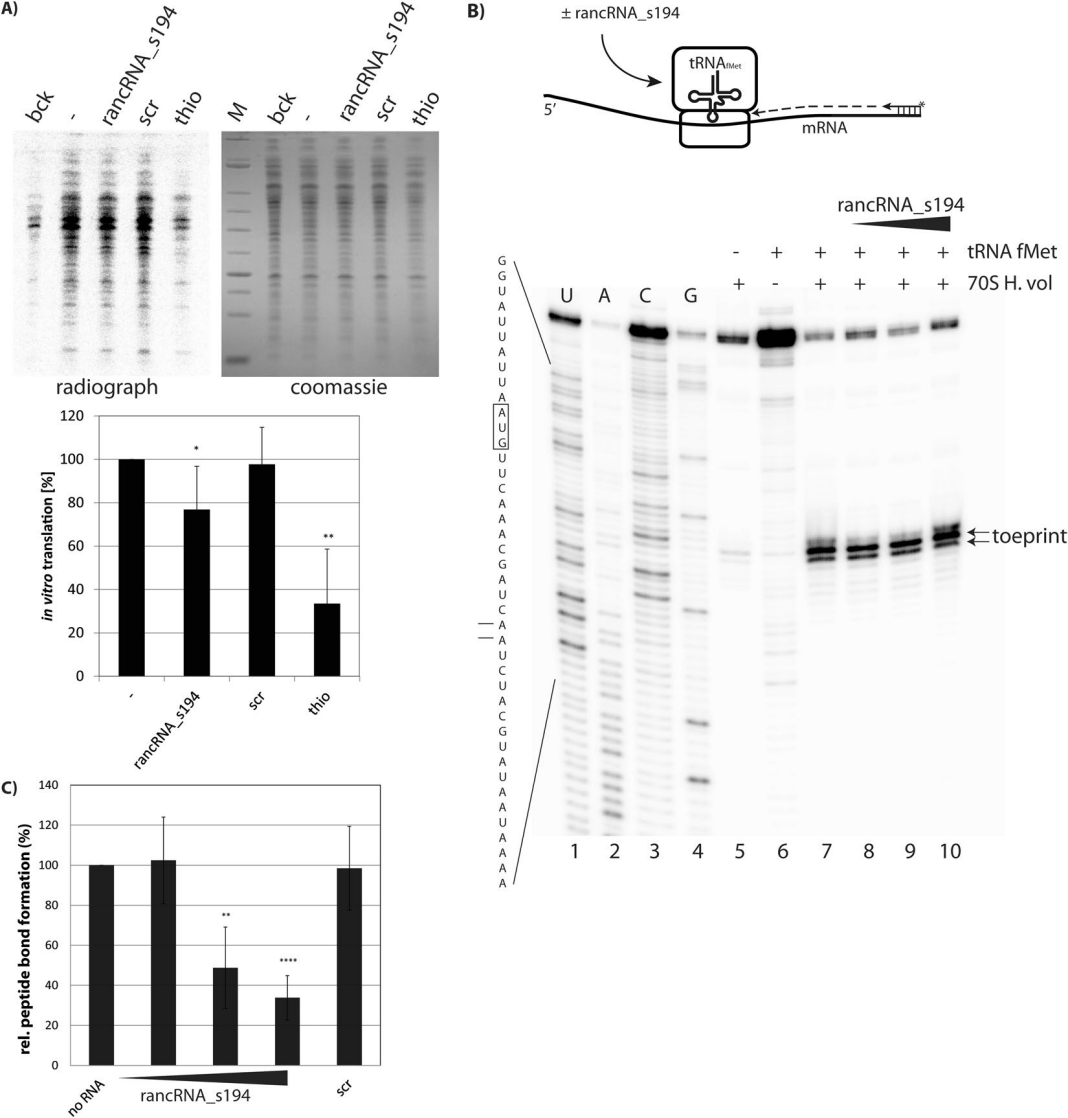
rancRNA_s194 does inhibit *in vitro* translation and peptide bond formation, but does not impair translation initiation complex formation. (**A**) *In vitro* translation with *H*. *volcanii* S30 cell extracts. Cell extracts were incubated with ^35^S-Methionine to label newly synthesized proteins which were visualized by SDS-PAGE and subsequent autoradiography. Reactions in the absence (−) or in the presence of rancRNA_s194, an RNA with the same length but randomized nucleotide sequence (scr), or the antibiotic thiostrepton (thio) were assembled. Additionally a complete *in vitro* translation reaction was incubated on ice (bck) and served as background signal, which was subtracted from all other samples. Lower panel shows mean and standard deviation of four independent experiments. (**B**) (top) Schematic representation of the toeprinting assay using *H*. *volcanii* 70S, mRNA (termed 8-codon-stop mRNA) and initiator tRNA^fMet^. Extension of a ^32^P-radiolabeled primer by reverse transcription (dotted arrow) is terminated in case of a stable 70S/mRNA/tRNA^fMet^ complex formation. (bottom) U, A, C, G Indicate dideoxy sequencing lanes (lanes 1–4). The relevant part of the mRNA sequence is shown on the left of the gel. The toeprinting site and the start codon are highlighted. The toeprinting signal depends on the simultaneous presence of 70S ribosomes and tRNA^fMet^ (lanes 5–7) and is unaffected by increasing amounts of rancRNA_s194 (lanes 8–10). The full-length gel is presented in Supplementary Fig. 9. (**C**) Peptide bond formation was assayed via the puromycin reaction. *H*. *volcanii* 70S ribosomes with P-site bound Ac[^3^H]Phe-tRNA^Phe^ were incubated with puromycin in the absence (no RNA) or in the presence of increasing amounts of rancRNA_s194. As specificity control a randomized RNA with same length (scr) was used which did not inhibit peptide bond formation at highest used concentrations. In (A) and (**C**) significant differences were determined using the 2-tailed paired Student’s t-test (***p < 0.001, **p < 0.01, *p < 0.05).

As the rancRNA_s194 was abundant in *H*. *volcanii* cells, showed strong association with actively translating ribosomes *in vivo* and showed binding to the large ribosomal subunit *in vitro* (Fig. 4), this ncRNA potentially could influence protein synthesis. To test this, *in vitro* translation reactions were assembled based on cell extracts isolated from the *H*. *volcanii* s194 knock-out strain (ΔrancRNA_s194). Translational activity was assessed by measuring ^35^S-Met incorporation into newly synthesized proteins in the absence or presence of *in vitro* transcribed rancRNA_s194. In the presence of rancRNA_s194 a very slight reduction of global protein synthesis was evident, while the scrambled control RNA had no effect (Fig. 5A). Reduction of *in vitro* protein synthesis in the presence of the antibiotic thiostrepton served as translational control. In principle every step of protein synthesis, namely initiation, elongation, termination and recycling, could be targeted by rancRNA_s194 resulting in the observed translation inhibition. Since the initiation is considered to be the rate limiting step of protein synthesis^18^, we first tested if rancRNA_s194 has an impact on the formation of an initiation complex. To do so we performed toeprinting experiments using *H*. *volcanii* ribosomes isolated from the ΔrancRNA_s194 strain, an mRNA analog carrying a unique AUG codon and deacylated tRNA^fMet^ (Fig. 5B)^14,19^. To assess if the 70 S/tRNA^fMet^/mRNA initiation-like complexes were formed, a reverse transcription inhibition-based toeprinting assay was performed in the absence or presence of rancRNA_s194. Successful establishment of the initiation-like complex is monitored by a specific toeprint at mRNA position +15/16 (the first nucleotide of the AUG start codon is assigned as position +1). Addition of increasing amounts of *in vitro* transcribed rancRNA_s194 did not influence complex formation and therefore does not seem to affect translation initiation (Fig. 5B). This interpretation is supported by the fact that rancRNA_s194 does neither compete with tRNA nor with mRNA (coding for r-protein L12 from *Methanococcus thermolithotrophicus*) for ribosome binding (Supplementary Fig. 4). This is unlike another functional rancRNA (Val-tRF) identified previously in *H*. *volcanii*, that strongly and globally interfered with initiation complex formation by competing with mRNA loading^14^. These data are compatible with two scenarios: either rancRNA_s194 inhibits a step downstream of the initiation phase of protein biosynthesis, or it affects recruiting of specific mRNAs to the initiating ribosome *in vivo*.

After initiation, the first peptide bond formed represents the entry of protein synthesis into the elongation phase. To gain insight into this step, the peptidyl transferase activity was assessed by means of the puromycin reaction. N-acetyl-[^3^H]Phe-tRNA was used as donor substrate and the antibiotic puromycin as acceptor for the transpeptidation reaction. Addition of rancRNA_s194 reduced peptide bond formation in a dose-dependent manner, whereas the scrambled control RNA had no effect (Fig. 5C), thus highlighting the specificity of the interaction. To gain *in vivo* insight into rancRNA_s194 action we used a previously established metabolic labeling assay which is based on the PEG-mediated transformation of synthetic rancRNAs into *H*. *volcanii* cells^14^. As can be seen in Supplementary Fig. 5, rancRNA_s194 does not affect metabolic labeling, whereas the antibiotic puromycin or the previously characterized tRNA-derived rancRNA (Val-tRF) inhibited protein production *in vivo*. These results indicate that either the amount of introduced rancRNA_s194 is too small to elicit detectable effects on global protein synthesis, or that rancRNA_s194 targets and inhibits only a subset of specific mRNAs *in vivo*.

### *H*. *volcanii* cells lacking rancRNA_s194 grow faster in xylose medium

**Figure 6:**
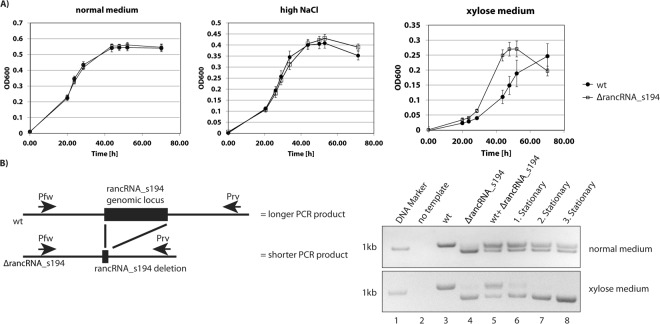
The rancRNA_s194 deletion strain shows a shortened lag phase and increased fitness in xylose-containing medium. (**A**) Growth curves in either normal medium, medium containing high NaCl (4 M) or synthetic medium containing only xylose as carbon source. (**B**) Growth competition experiments with *H*. *volcanii* wildtype and rancRNA_s194 knock-out cells. An equal mixture of both strains was inoculated in the same flask and grown in either normal medium or synthetic xylose medium to the stationary phase three times. Genomic DNA was isolated after each stationary phase and the ratio between rancRNA_s194 knock-out and wildtype cells was determined by PCR. The left panel shows a schematic representation of the PCR setup. On the right an agarose gel shows the PCR products obtained at the onset of the growth competition experiment (lane 5) and after each stationary phase (lanes 6–8) (expected size of PCR products: wt, 1,018 nucleotides; knock-out, 901 nucleotides). The full-length agarose gels are presented in Supplementary Fig. 9.

To investigate the biological importance of rancRNA_s194 we analyzed growth of *H*. *volcanii* wildtype cells compared to the knock-out strain during different growth conditions. In most of the tested conditions both strains had comparable growth characteristics (Fig. 6A and Supplementary Fig. 6). The strongest phenotype was evident in a synthetic medium with xylose as the only carbon source. Under these incubation conditions the rancRNA_s194 knock-out strain had a shorter lag phase compared to wildtype strain, however the cell densities reached in stationary phase were identical (Fig. 6A). Similar observations with this strain were made before by others^9,17^. To assess possible fitness differences of the knock-out strain compared to the parental wildtype cells, a growth competition experiment was performed. *H*. *volcanii* wildtype cells and rancRNA_s194 deletion cells were mixed together in equal amounts and this mixture was then used for inoculating either normal medium or synthetic medium containing xylose as sole carbon source. The cells were grown into the stationary phase and then re-diluted to inoculate fresh medium. This procedure was done in total three times. Genomic DNA (gDNA) was isolated after each stationary phase and the fraction of each of the two strains within the mixture was determined by PCR. Since the PCR product obtained from the knock-out strain is shorter than the one obtained from wildtype gDNA (Fig. 6B), any growth competition can be monitored by this experimental set up. In normal medium, both strains grew uniformly and the ratio did not change during the three growth cycles (Fig. 6B). This indicates that lack of rancRNA_s194 does not pose a fitness penalty in rich media. However, in the synthetic xylose medium the rancRNA_s194 deletion strain completely overgrew the parental *H*. *volcanii* cells already after the first growth cycle (Fig. 6B). These data fully confirm the growth curves above and suggests that rancRNA_s194 plays a physiological role in case non-standard sugars, such as xylose, need to be metabolized.

### rancRNA_s194 acts as translational regulator of a specific mRNA involved in sugar metabolism

As rancRNA_s194 RNA seems to regulate growth of *H*. *volcanii* cells in synthetic xylose-containing medium (Fig. 6), we wanted to investigate if association of the rancRNA to ribosomal particles differs in cells grown under these conditions compared to cells grown in normal medium. Polysome profiling followed by northern blot analysis, however, revealed no differences of rancRNA_s194 association to ribosomal particles (Supplementary Fig. 7).

**Figure 7:**
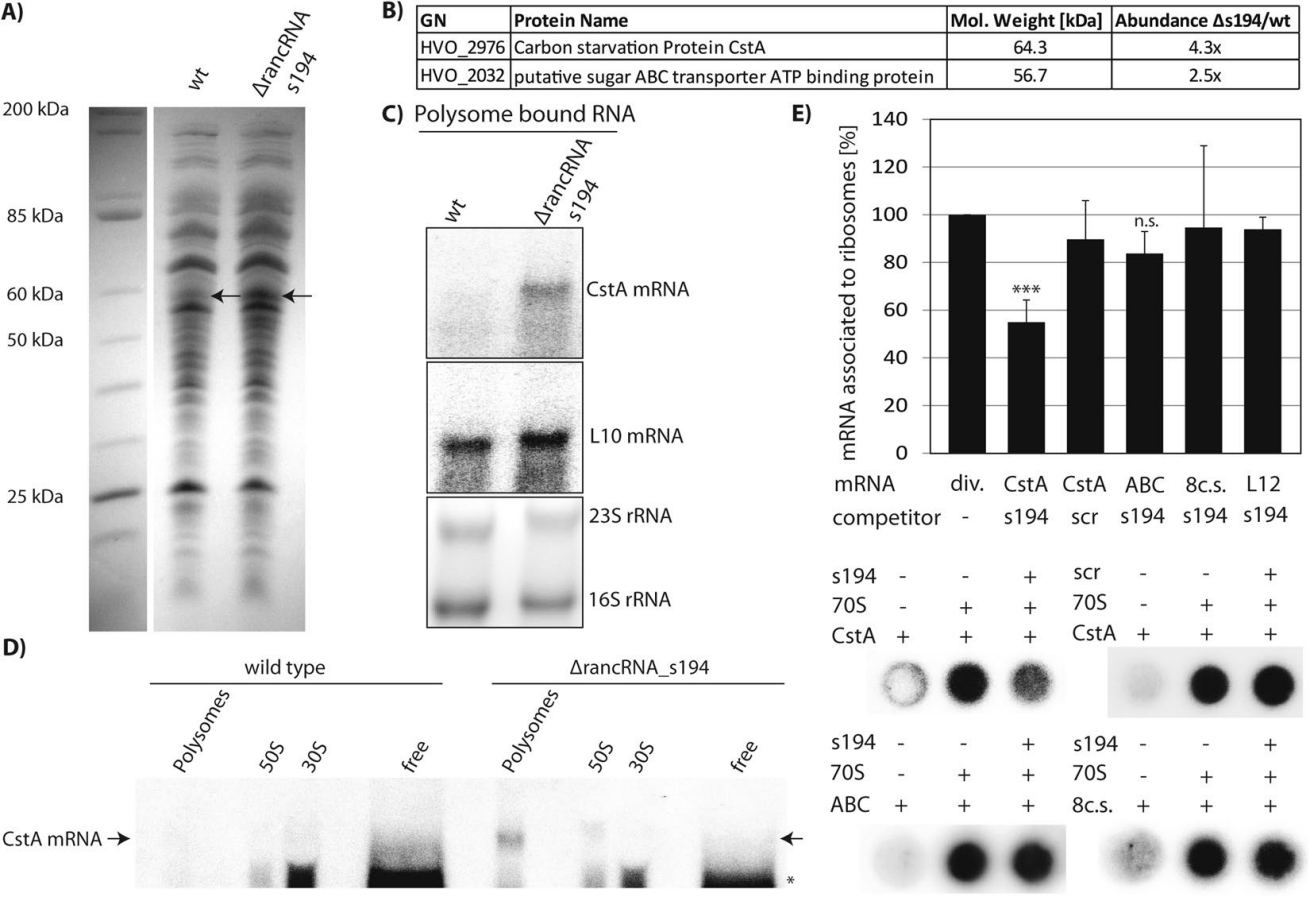
Differential mRNA translation between wildtype and rancRNA_s194 deletion strains in xylose medium. (**A**) Cellular protein extracts (S30) were prepared from *H*. *volcanii* wildtype (wt) and rancRNA_s194 knock-out (Δ) cells inoculated in xylose medium for the same time period. Proteins were separated on an 11% SDS-tricine gel and stained with coomassie. The arrow indicates the position of a pronounced protein band seen in the knock-out strain. The marker lane and the two experimental lanes were cropped from different parts of the same SDS gel. (**B**) Two most strongly upregulated proteins in the rancRNA_s194 knock-out strain compared to wildtype *H*. *volcanii* as identified by mass spectrometry. (**C**) Northern blot analysis of the carbon starvation protein *cstA* mRNA and mRNA coding for ribosomal protein L10. mRNAs were isolated from the polysomal fraction of sucrose gradients of the wt or the ΔrancRNA_s194 *H*. *volcanii* cells grown in xylose-containing medium for the same time. Ethidium bromide-stained 23S rRNA and 16S rRNA (lower gel) served as loading controls. Full-length blots are presented in Supplementary Fig. 9. (**D**) The distribution of the *cstA* mRNA between different polysome gradient fractions was monitored by northern blot analysis and compared between wildtype and rancRNA_s194 knock-out *H*. *volcanii* cells. In all gradient fractions corresponding volumes were loaded, except for the light gradient fraction (free), where only 50% of the corresponding volume fraction was applied. The asterisk indicates unspecific annealing of the northern blot probe (see Supplementary Fig. 10 for uncropped blots and the ethidium bromide stained gel). (**E**) Binding of radiolabeled mRNAs (CstA; sugar ABC transporter ATP binding protein, ABC; 8-codon-stop mRNA, 8 c.s.; r-protein L12, L12) to *H*. *volcanii* ribosomes was measured by filter binding in the absence (−) or presence of unlabeled competitor rancRNA_s194, or the scrambled version thereof (scr). The mean and standard deviation of three to six independent filter binding experiments is shown. Signals measured in the absence of ribosomal particles were subtracted from all experimental points. The binding data with r-protein L12 mRNA have been performed twice and are identical to the ones shown in Supplementary Fig. 4B. Significant differences were determined using the 2-tailed paired Student’s t-test (***p < 0.001, n.s., not significant). Phosphorimager screens of representative filter binding competition experiments are shown.

Since rancRNA_s194 seems to modulate protein biosynthesis, we next investigated its role in shaping the proteome. To get a first hint if the protein content of *H*. *volcanii* ΔrancRNA_s194 cells differs from the wildtype strain in xylose-containing medium, protein extracts from both strains were separated on denaturing SDS polyacrylamide gels. While most of the visible bands were identical in both strains, one prominent protein band in the size range of about 55–65 kDa was more abundant in the knock-out strain compared to the wildtype cells (Fig. 7A). The band was excised from the gel and the protein content analyzed by mass spectrometry. From the obtained list of protein candidates, two were in the size range of interest and significantly more abundant in the gel slice obtained from the rancRNA_s194 knock-out cells. One was the carbon starvation protein (CstA) and the other one a putative sugar ABC transporter ATP-binding protein. According to the mass spectrometric analyses these two proteins were 4.3 and 2.5 fold more abundant, respectively, in the ΔrancRNA_s194 cells (Fig. 7B). To verify these predictions, northern blot analysis on mRNAs isolated from the polysome fraction of wildtype or knock-out cells during exponential growth in xylose medium was performed. Using radiolabeled probes targeting the mRNA of these two protein candidates clearly showed that the *cstA* mRNA associates only with polysomes in cells that lack rancRNA_s194 (Fig. 7C). Northern blots aimed at identifying the mRNA of the putative sugar ABC transporter ATP-binding protein did not show any signal. As a house keeping control, targeting the mRNA coding for the ribosomal protein L10 on the other hand showed essentially equally strong northern blot signals in both the wildtype and the rancRNA_s194 deletion strain. Polysome profiling and monitoring *cstA* mRNA distribution in the different gradient fractions in wildtype and rancRNA_s194 knock-out cells corroborate these findings and shows *cstA* mRNA signals in the actively translating polysomes solely in the rancRNA deletion strain (Fig. 7D). To gain mechanistic insights of rancRNA_s194 action on the ribosome, filter binding competition experiments with various mRNAs were performed. While rancRNA_s194 was capable of efficiently competing with the *cstA* mRNA for ribosome binding, all other tested mRNAs (including the one coding for the putative sugar ABC transporter ATP-binding protein) failed to be removed by rancRNA_s194 (Fig. 7E). Importantly, *cstA* mRNA was also not removed from the *H*. *volcanii* ribosome by the scrambled version of rancRNA_s194. All these findings are compatible with the view that rancRNA_s194 regulates protein synthesis of CstA during growth in xylose-containing medium by preventing its translation during the exponential phase, when rancRNA_s194 is most abundant.

## Discussion

Ribosome-associated ncRNAs (rancRNAs) are an emerging class of ribo-regulators of protein biosynthesis in all domains of life and encompass small as well as long ncRNAs^11^. In this study we analyzed the small RNA interactome of ribosomes in the halophilic archaeon *H*. *volcanii* under various growth conditions. Bioinformatics analyses revealed 1,212 putative rancRNA candidates (Fig. 1). We showed that an intergenic rancRNA, called rancRNA_s194, is expressed in the exponential growth phase under all tested stress conditions and associates primarily with polysomes (Figs 3 and 4). The binding site of rancRNA_s194 is located on the large ribosomal subunit and its occupancy results in inhibition of peptide bond formation and translation *in vitro* (Figs 4 and 5). Genomic deletion of rancRNA_s194 yields a *H*. *volcanii* strain that possesses a shortened lag phase in media containing xylose, or other non-standard sugars, as sole carbon source (Fig. 6 and Supplementary Fig. 6). This gain of function behavior allows the knockout strain to readily outcompete the wildtype strain in a growth competition experiment in xylose-containing medium. Proteome analyses and polysome profiling revealed the carbon starvation protein *cstA* mRNA as prime target of rancRNA_s194 translation regulation (Fig. 7). Such gain of function phenotypes in small ncRNA knock-out strains have been reported before in archaea and eukarya^9,20^. This indicates that ncRNA-controlled metabolic pathways did not necessarily evolve to guarantee maximal growth rates, rather than to allow the establishment of a robust and tunable network capable of adjusting gene expression to rapidly changing environmental conditions.

In variance to our recently identified mRNA- and tRNA-derived rancRNAs (rancRNA_18 and Val-tRF, respectively) that were demonstrated to inhibit translation on a global scale^12–14^, rancRNA_s194 is an mRNA-specific regulator. This interpretation is supported by the findings that rancRNA_s194 did not affect overall translation *in vivo* as assessed by metabolic labeling in *H*. *volcanii* or in yeast (Supplementary Fig. 5). In contrast, the rancRNAs that were shown to inhibit global translation (rancRNA_18, ref.^13^; Val-tRF, ref.^14^) were also effective in heterologous translation systems. Only under *in vitro* conditions, where the system can be overloaded with synthetic rancRNA_s194, we do observe global inhibition of *in vitro* translation and peptide bond formation (Fig. 5A,C). Over the years it has been revealed that the ribosome is a highly dynamic RNA-protein particle. Accumulating biochemical as well as structural evidence suggests that this dynamic behavior is a prerequisite for efficient protein biosynthesis^21^. Thus it is possible that the inhibitory effects seen in the puromycin reaction *in vitro* might originate from high levels or ribosome-bound rancRNA_s194 blocking the ribosome in its intrinsic movements. *In vivo* the concentration of rancRNA_s194 (~2,000 copies per *H*. *volcanii* cell; Supplementary Fig. 8) does not seem to be sufficiently high to affect global translation of all mRNAs (Supplementary Fig. 5A). However, in the absence of rancRNA_s194 the *cstA* mRNA appears to be preferentially translated during growth in xylose medium (Fig. 7). In bacteria the membrane associated protein CstA is proposed to be involved in starvation response as a peptide transporter^22–24^. CstA is highly upregulated under nutrient limiting conditions during stationary phase compared to exponential phase cultures in *Campylobacter jejuni*^25^. CstA expression in bacteria is regulated on the translational level by the RNA binding carbon storage regulator A protein (CsrA) which binds to the Shine-Dalgarno sequence on the *cstA* mRNA preventing binding of the initiating ribosome^26^. The CstA protein is highly conserved in bacteria and can be found in 1,649 species from 20 different phyla^27^. Furthermore CstA is found in 55 archaeal species, including *H*. *volcanii*. In contrast, the regulatory CsrA protein, which is highly conserved in bacteria, was only found in one archaeal organism, but not in *H*. *volcanii*. Since the *cstA* mRNA in *H*. *volcanii* has a very short 5′-UTR of 13 nucleotides lacking a canonical Shine-Dalgarno sequence^5^, other mechanisms of translation regulation are expected to be at work. In fact it has been reported that *H*. *volcanii* is an archaeal species which does not make use of the Shine-Dalgarno mechanism for translation initiation^28^. Thus it is plausible that rancRNA_s194 took over the CstA translation regulation role in *H*. *volcanii*. Our data suggest that rancRNA_s194 is a reasonable candidate for repressing the *cstA* mRNA during the elongation phase in a not yet fully understood mRNA-specific manner (Fig. 7E). By applying the target prediction program IntaRNA^29^ a stretch of eight nucleotides complementarity between rancRNA_s194 and the *cstA* mRNA (from position 61–68 downstream of the AUG start codon) was identified. The eight residues on rancRNA_s194 that are involved in this predicted interaction all reside in a loop region of the secondary structure (Fig. 2D) and thus resemble known prokaryal sRNA-mRNA type of targeting mechanisms^30^. Why rancRNA_s194 needs to interact with the ribosome in order to fulfill this mRNA-specific translation regulation role awaits further investigations. The data shown in Fig. 7D reveal that overall *cstA* mRNA levels are lower in the exponential wildtype cells as compared to the rancRNA_194 deletion strain. This is likely due to the fact that in the presence of rancRNA_s194 the *cstA* mRNA is excluded from the pool of translating ribosomes and thus more prone to rapid degradation. Alternatively rancRNA_s194 might also regulate the transcription of *cstA* mRNA in *H*. *volcanii*. However, the almost exclusive ribosome-association of this ncRNA in the cell (Fig. 4 and Supplementary Fig. 7) and its capability to remove *cstA* mRNA specifically from the ribosome (Fig. 7E), argue for a role of rancRNA_s194 during translation control. rancRNA_s194 is primarily expressed and ribosome-bound during exponential growth where CstA functions are likely not needed. However upon entry into stationary phase the cellular levels of rancRNA_s194 drop significantly which would alleviate translation repression of CstA. Knock-out of the rancRNA_s194 gene locus in *H*. *volcanii* resulted in the pronounced appearance of a protein band on denaturing gels already during exponential growth, which might correspond to CstA (Fig. 7A). Since CstA is reported to be a peptide permease and thus involved in peptide uptake, it appears likely that the rancRNA_s194 knock-out strain has the capability of metabolizing peptides and amino acids already during exponential growth.

The genomic locus of rancRNA_s194 possesses all hallmarks of a genuine gene, namely it is independently transcribed, contains a TATA box in an optimal position 28 nucleotides upstream of the transcription start site^5^, and its expression is also differentially regulated during growth (Fig. 3A). The rancRNA_s194 biology seems to focus on orchestrating a very specific stress response in *H*. *volcanii* cells. In fact the corresponding gene is conserved only in eight other Haloferax species with more than 94% sequence identity. Gene regulation by rancRNAs has the principal advantage of enabling a swift reaction to environmental stimuli, since the regulatory molecule is a short RNA that does not need to be translated to fulfill its regulatory potential. Such rapid control mechanisms are particularly important for prokaryal organisms where regulatory effects are often observed within minutes, therefore allowing fast adaption to new challenges^31^. Compared to other so far functionally characterized small rancRNAs^12–14^, rancRNA_s194 is special since (i) it is encoded as separate gene (see above) and (ii) regulates translation of only a subset of, or even an individual, mRNA. In that sense rancRNA_s194 resembles other mRNA-specific ribosome-bound ncRNAs such as the bacterial tmRNA or the signal recognition particle RNA present in all domains of life. The data presented herein add another rancRNA to this recently detected class of regulatory molecules and reveal yet another layer of gene regulation on the post-transcriptional level.

## Materials and Methods

### Strain and growth conditions

*Haloferax volcanii* strain H26 was used as “wild type” and a knock out strain of the s194 RNA genomic locus in the genomic background of strain H26 (Δs194)^17^. Normal growth conditions were as described before^14^. For stress conditions concentrations of individual components were changed to 1.5 M NaCl for low NaCl stress, 4 M NaCl for high NaCl stress, 450 mM MgSO_4_ for high Mg^2+^ stress. Cold shock was performed in normal medium at 30 °C. For high pH stress 100 ml 1 M Tris-HCl pH 8.5 were added to one liter complex medium. For synthetic medium 900 ml salt solution were prepared with 2.1 M NaCl, 220 mM MgCl_2_*6 H_2_O, 41 mM MgSO_4_*7 H_2_O, 13 mM KCl, 9 mM CaCl_2_, 50 mM Tris-HCl (pH 7.2) in dH_2_O and the solution was autoclaved. Then the following components were added to final concentration of 1 mM K_2_HPO_4_, 10 mM NH_4_Cl, 50 μg/ml uracil, 1.8 μM MnCl_2_*4 H_2_O, 1.5 μM ZnSO_4_*7 H_2_O, 8.2 μM FeSO_4_*7 H_2_O, 0.2 μM CuSO_4_*5 H_2_O and 1 ml BME Vitamins 100 x solution (B6894 *Sigma*). As carbon source xylose was added to 0.5% (w/v) and the solution was then filled up to the end volume of 1 l with distilled H_2_O. Growth curves were performed in 96 well plates as described^17^. Per condition three biological replicates per strain and seven technical replicates per biological replicate were measured.

### RancRNA library preparation and analysis of deep sequencing results

cDNA library encoding ribosome-associated RNA in the size range between 20–500 nucleotides was prepared and deep-sequenced as described previously^12,15,32^. The obtained sequence reads have been deposited in ENA (Acc. No PRJEB25902). Reads were analyzed using the APART pipeline^15^. From the initially obtained 73.5 Mio raw reads only those with a minimal length of 18 nucleotides were further analyzed which also contained both the 5′ and the 3′ adaptors. The adaptors were then removed from the sequence reads and reads were mapped to the *H*. *volcanii* genome (*Haloferax volcanii* DS2 Mar 30 2010 Assembly (haloVolc1)). Only one mismatch was allowed for mapping the sequences to the genome. Overlapping reads were assembled into contigs and stable RNA species were identified (revealed by a substantial change in read coverage at 5′ and 3′ ends (for details see ref.^15^)).

### Isolation of ribosomes, ribosomal subunits and polysome gradients

*H*. *volcanii* cells grown to exponential growth phase were harvested by centrifugation. The cell pellet was resuspended in buffer RI (3 M KCl, 150 mM MgCl_2_, 6 mM β-mercaptoethanol, 10 mM Tris/HCl pH 7.6, 0.5 mM DTT) and the cells were opened with a French press or with a mortar as described previously^12^. The cell debris was removed by centrifugation (30,000 *× g*, 15 min). The supernatant was treated with RNase-free DNase (10 min on ice) and subjected to a second centrifugation (30,000 *× g*, 15 min) resulting in an S30 cell extract. The lysate was then layered on a 1.1 M sucrose cushion and ribosomes were pelleted with ultracentrifugation at 100,000 *× g* at 4 °C for 17 h in a Ti-60 Beckman rotor resulting in a crude ribosome pellet (P100). The ribosomal pellet was resuspended in buffer Pu (3 M KCl, 70 mM Hepes/KOH (pH 7.8), 60 mM Mg(OAc)_2_, 6 mM β-mercaptoethanol). The supernatant of the 100′000 *× g* centrifugation step (S100) was used for RNA isolation by precipitation with 2.5 eq. ethanol followed by PCI extraction. For isolation of ribosomal subunits, the ribosomal pellet (P100) was dissolved in dissociation buffer (2.7 M KCl, 450 mM NH_4_Cl, 10 mM MgCl_2_, 20 mM Tris/HCl (pH 7.6), 6 mM β-mercaptoethanol) and the samples were then dialyzed twice against 400 ml dissociation buffer at 4 °C using a Spectra/Por cellulose ester membrane with a molecular weight cut off of 3,500 Dalton. 200 OD_260_ of the sample was then layered on a linear sucrose gradient (10–40%) prepared in dissociation buffer, and ultracentrifugation was performed at 25,000 rpm for 15 h at 4 °C in a SW28 Beckman rotor. The gradient was then pumped out using a peristaltic pump while measuring OD_254_ and fractions containing 50 S or 30 S ribosomal subunit were collected. The magnesium concentration was then adjusted to 150 mM and ribosomal subunits were pelleted at 100,000 *× g* at 4 °C for 17 h in a Ti-60 Beckman rotor. To obtain highly pure ribosomal subunits, the above described procedure was repeated and finally ribosomal subunits were dissolved in buffer Pu. Polysome gradients were performed as described^12^.

### RNA isolation and northern blot analysis

*H*. *volcanii* total RNA was isolated using Tri-reagent (*Sigma Aldrich*) according to the manufacturer’s instructions. Ribosome-associated RNA was isolated from crude ribosomal pellets (P100) by PCI extraction. The RNA not associated with ribosomes was isolated from the supernatant after centrifugation at 100,000 × g (S100) by precipitation with 2.5 eq. ethanol followed by PCI extraction. For RNA associated with either polysomes, 50 S or 30 S ribosomal particles, fractions collected after the polysome gradient were dialyzed twice against 1,000 ml pure distilled water (30 min, 4 °C, Spectra/Por cut off of 3,500 Dalton) followed by ethanol precipitation. Northern blot analysis was performed as described in ref.^12^ using 10–20 μg of RNA. The following DNA oligonucleotides were used: rancRNA_s194NB, Hts4NB and IG1.7NB (for primer sequences see Supplementary Table 1). For the mRNA northern blots, 3.2 µg polysomal RNA was separated on a 1.4% agarose gel in 1x MOPS and 0.2 M formaldehyde and transferred by passive capillary blotting overnight at 4 °C. For northern blot analyses of all polysome gradient fractions, 5 µg of polysomal RNA was loaded on an 1.4% agarose gel and the corresponding equal volumes of the 50S and 30S subunit fractions were applied. Due to the significantly larger volume of the “free RNA” (corresponding to the light gradient fraction) only half of the equivalent volume was loaded on the gel. Probes for the L10 ribosomal protein mRNA, the *cstA* and the ABC transporter ATP binding protein mRNA were made by amplifying part of the genes by PCR (primers L10 fw and L10rv, CstA_T7_fw and CstA_T7_rv or 2032ABCfw, 2032ABCrv, respectively) followed by making multiple radioactively labeled probes by extending random hexamer primers with the Klenow fragment in the presence of α-^32^P-dCTP. Probes were then hybridized overnight at 65 °C in hybridization buffer containing 0.5 M Na_2_HPO_4_, 7% w/v SDS, 1% w/v bovine serum albumin and 0.9 mM EDTA. After hybridization, the blots were first washed in pre-warmed 2x SSC with 0.1% w/v SDS for 30 min and then washed again using 0.2x SSC with 0.1% SDS. Alternatively for detection of *cstA* mRNA the DNA oligonucleotides CstA1NB and CstA2NB were used (Supplementary Table 1) and hybridization was performed overnight at 42 °C as described^12^.

### RNA *in vitro* transcription

*In vitro* transcription reactions were performed as described previously^33,34^. PCR primers used for making the s194 DNA template from genomic DNA were s194fw(T7) and s194rv (Supplementary Table 1). For the scrambled (scr) RNA control, two overlapping synthetic DNA oligos with a randomized sequence after the T7 promotor were designed and the DNA template was generated by PCR with the overlapping oligos scr1T and scr2T. In a second step, on this initial PCR product, a second PCR was performed with primers scr1T and scr(rv) (Supplementary Table 1**)**, resulting in a construct of the same length as the rancRNA_s194 RNA but with randomized nucleotide sequence. To transcribe parts of the L10 ribosomal protein mRNA (first 491 nt), the carbon starvation protein *cstA* mRNA (first 511 nt) and the ABC transporter ATP binding protein mRNA (first 997nt), the DNA template for T7 transcription was generated by PCR on gDNA using the following primer pairs: L10_T7_fw, L10_T7_rv; CstA_T7_fw, CstA_T7_rv; Hvo_2032_T7fw, Hvo_2032_T7_rv **(**Supplementary Table 1**)**.

### Filter binding assay

All filter binding assays were performed as described previously^14^. For mRNA-rancRNA competition experiments, radioactively labeled *in vitro* transcribed mRNA (4 pmol) and the unlabeled competitor rancRNA (8 pmol) were added before addition of *H*. *volcanii* 70 S ribosomes (5 pmol).

### *In vitro* translation

The *H*. *volcanii* rancRNA_s194 knock-out strain was grown to the exponential phase (OD_600_~0.8), harvested and resuspended in Buffer P (3.4 M KCl, 100 mM Mg(OAc)_2_, 6 mM 2-mercaptoethanol, 10 mM Tris-HCl pH 7.6). Cells were opened with a French press and the cell debris was removed as described above. The resulting S30 cell extract was then used for *in vitro* translation by incubating 11.4 µl with 0.6 µl ^35^S-Met for 1 h at 42 °C either in the absence or presence of 300 pmol *in vitro* transcribed rancRNA candidates (final volume 15 µl). The reactions were stopped by directly applying to G25-spehadex mini columns and subsequently the proteins were separated on 11% tricine-SDS gels. Proteins were stained with coomassie-brilliant blue and the newly synthesized proteins were monitored by phosphor imaging (Typhoon FLA-9000, Fujifilm) and quantified with IQTL 8.1.

### Toeprinting analysis

Identification of an initiation-like complex composed of 70S ribosomes, mRNA (termed 8-codon-stop mRNA) and tRNA^fMet^ was performed as described^14^. In brief, 15 pmol of crude *H*. *volcanii* 70S particles (from P100) isolated from the ΔrancRNA_s194 strain were incubated in the presence of 100, 200 or 300 pmol *in vitro* transcribed rancRNA_s194.

### Peptidyl transferase assay

The puromycin reaction was used to assess peptide bond formation as described before^14^. In brief, the P-sites of 10 pmol *H*. *volcanii* 70S ribosomes, isolated from the ΔrancRNA_s194 strain, were bound with 0.8 pmol N-acetyl-[^3^H]Phe-tRNA^Phe^ and incubated with *in vitro* transcribed rancRNAs (0, 10, 50, 100 pmol) and 2 mM puromycin. In addition to the components described in ref.^14^, poly(U) RNA was added to a final concentration of 2 μg/ul.

### Growth competition

*H*. *volcanii* wildtype and ΔrancRNA_s194 cells were inoculated in equal amounts into the same flask containing either normal medium or synthetic medium with xylose as sole carbon source. Cells were then grown to stationary growth phase and from this culture, new medium was inoculated and this procedure was repeated two more times. Genomic DNA was isolated before strains were mixed, after mixing and when the cultures reached the stationary phase. Genomic DNA was purified by PCI extraction (pH 8) and the PCR was performed on the genomic DNA to identify the ratio between the wildtype and the ΔrancRNA_s194 strain. PCR was performed with the Phusion High-Fidelity DNA Polymerase (*Thermo Scientific*) with GC Buffer according to the manufacturer’s manual. Used PCR primers were s194gc(fw) and s194gc(rv) (Supplementary Table 1**)**.

### Protein analysis by mass spectrometry

Proteins were isolated as described above (S30 preparation) from wt or ΔrancRNA_s194 *H*. *volcanii* cells which were grown for the same time in xylose-containing synthetic medium. Protein concentration was measured with a nanodrop apparatus and proteins were then separated on an 11% SDS-tricine gel and stained with coomassie brilliant blue. For Mass spectrometry, slices of equal size were cut out and proteins were quantified by label free LC-MS/MS at the Mass Spectrometry facility of the University of Bern. Samples were isolated from three independent experiments and with two technical replicates. The quantification method Top3 (ref.^35^) was applied to determine the protein abundance and compared between the wt and ΔrancRNA_s194 samples.

## Electronic supplementary material

Supplementary data
