# Supplementary material for: mRNA-specific translation regulation by a ribosome-associated ncRNA in Haloferax volcanii
Source: Sci Rep. 2018 Aug 21;8:12502. doi: 10.1038/s41598-018-30332-w (PMC6104027; doi:10.1038/s41598-018-30332-w)
Supplement: Supplementary file 1 — Supplementary data [file 41598_2018_30332_MOESM1_ESM.pdf]

SUPPLEMENTARY DATA

mRNA-specific translation regulation by a ribosome-associated ncRNA in *Haloferax volcanii*

Leander Wyss[1,2], Melanie Waser[1], Jennifer Gebetsberger[1#], Marek Zywicki[3] and Norbert Polacek[1]*

[1]Department of Chemistry and Biochemistry, University of Bern, Freiestrasse 3, 3012 Bern, Switzerland

[2]Graduate School for Cellular and Biomedical Sciences, University of Bern, Bern, Switzerland

[3] Institute of Molecular Biology and Biotechnology, Adam Mickiewicz University, Umultowska 89, 61-614 Poznan, Poland

[#]current address: Institute of Organic Chemistry and Center for Molecular Biosciences (CMBI), Leopold-Franzens University, Innsbruck, Austria

*To whom correspondence should be addressed. Tel: +41 316314320; Email: norbert.polacek@dcb.unibe.ch

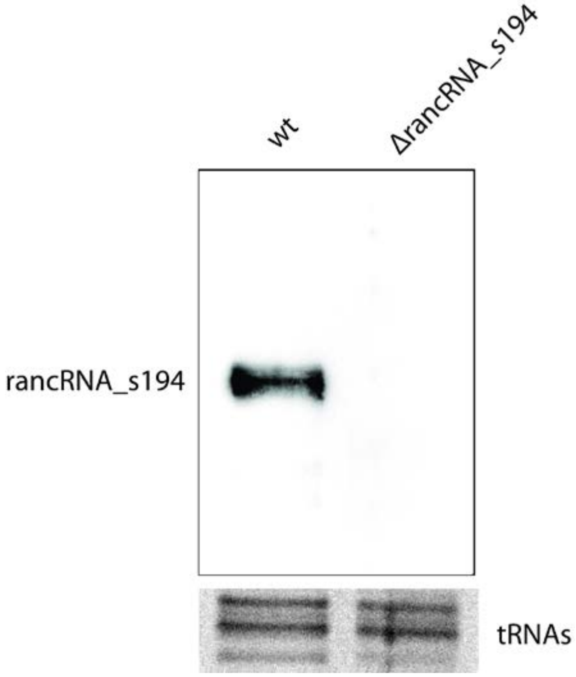

Supplementary Fig. 1. Confirmation of the rancRNA_s194 genomic knock out**.** Northern blot analysis with a radiolabeled DNA probe complementary to rancRNA_s194 confirmed the knock-out of the corresponding gnomic loci. Total RNA was either isolated from *H. volcanii* wildtype (wt) cells or from the knock-out strain(ΔrancRNA_s194)[1]. Ethidium bromide-stained tRNAs served as loading control.

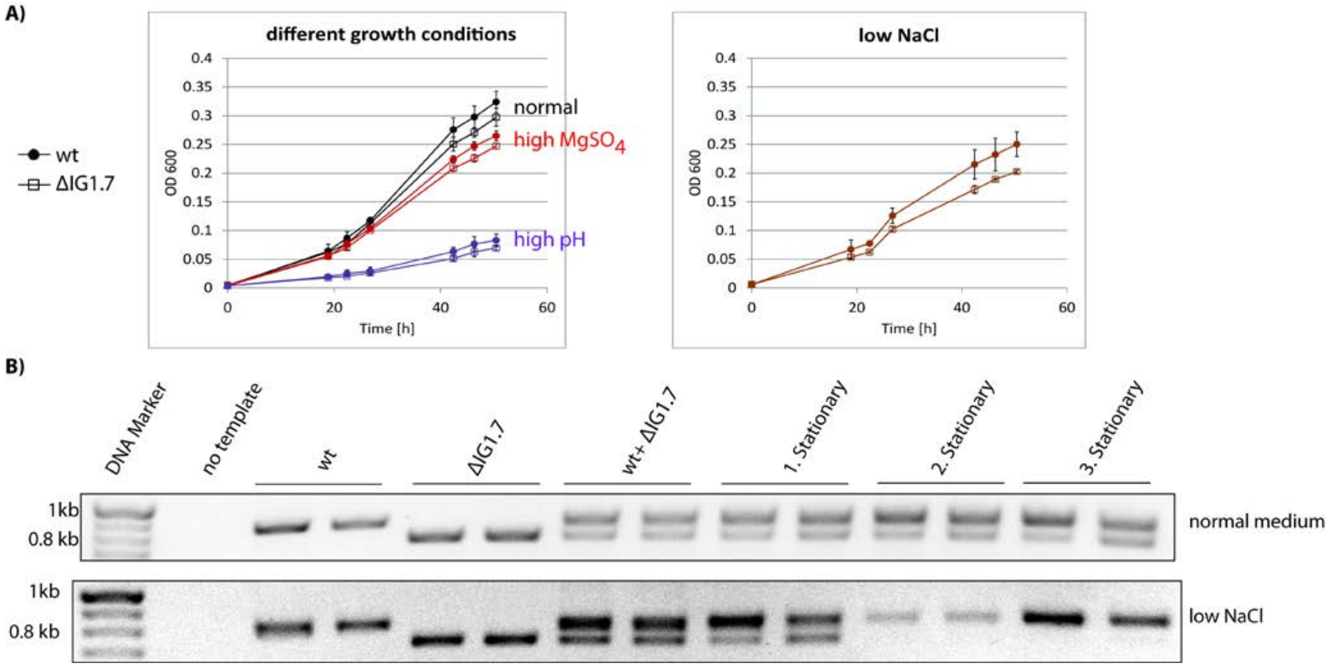

Supplementary Fig. 2. The ncRNA candidate IG1.7 plays a biological role in low salt stress. **(A)** Growth curves of *H. volcanii* wildtype (wt) or IG1.7 knock-out (ΔIG1.7) cells were determined in either normal medium, at high $MgSO_4$ concentration or with an alkaline pH of 8.5 (left graph) or in medium containing low NaCl concentration of 1.3 M (right graph). The mean and the standard deviations of three biological and seven technical replicates are shown. **(B)** Growth competition experiments with *H. volcanii* wildtype and IG1.7 knock-out cells. An equal mixture of both strains was inoculated in the same flask and grown either in normal medium or in medium with lower NaCl concentration (1.5 M) to the stationary phase three times. Before dilution and re-inoculation into fresh medium genomic DNA was isolated after each stationary phase. The ratio between knock-out and wildtype cells was determined by PCR (expected size of PCR products: wt, 827 nucleotides; knock-out, 727 nucleotides). In low salt medium the IG1.7 strain is outcompeted by the wildtype cells after the first stationary phase. In all cases the PCR results from two biological replicates are shown.

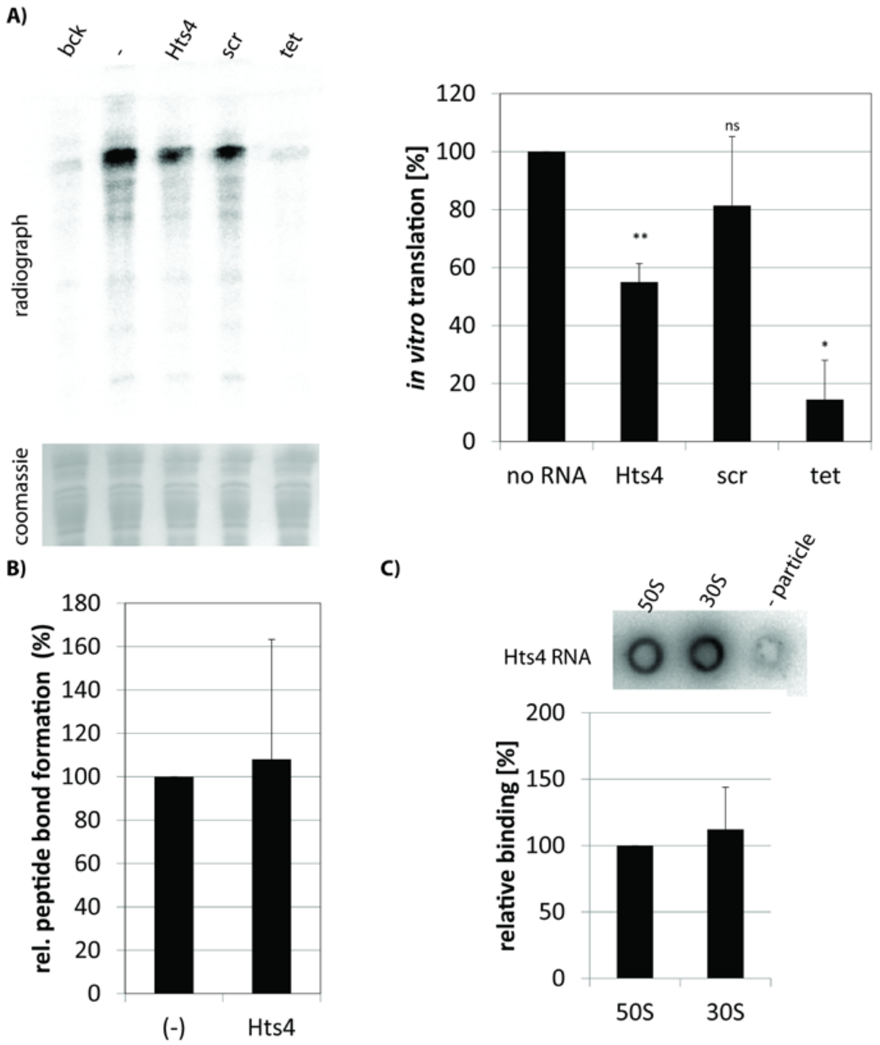

Supplementary Fig. 3. The ncRNA candidate Hts4 binds to ribosomes and inhibits translation. **(A)** *In vitro* translation with *H. volcanii* S30 cell extracts. Cell extracts were incubated with ^{35}S-Methionine to label newly synthesized proteins which were visualized by SDS-PAGE and subsequent autoradiography (left). Reactions in the absence (-) or in the presence of rancRNA candidate Hts4, an RNA with the same length but randomized nucleotide sequence (scr), or the antibiotic tetracyclin (tet) were assembled. Additionally a complete *in vitro* translation reaction was incubated on ice (bck) and served as background signal, which was subtracted from all other samples. Right panel shows mean and standard deviation of three independent experiments. Significant differences were determined using the 2-tailed paired Student's t-test (***p < 0.001, **p < 0.01, *p < 0.05). **(B)** Peptide bond formation in the absence (-) or presence of Hts4 RNA was assayed via the puromycin reaction. The mean and standard deviations of three reactions with ribosomes prepared from the *H. volcanii* Hts4 deletion strain are shown. **(C)** *In vitro* filter binding studies of [^{32}P]-radiolabeled *in vitro* transcribed Hts4 RNA on purified ribosomal subunits. Lower panel shows the mean and standard deviations of two experiments whereas binding of Hts4 RNA to 50S ribosomal subunit was set to 100 %. The signal measured in the absence of ribosomal subunits (-particle) was always subtracted.

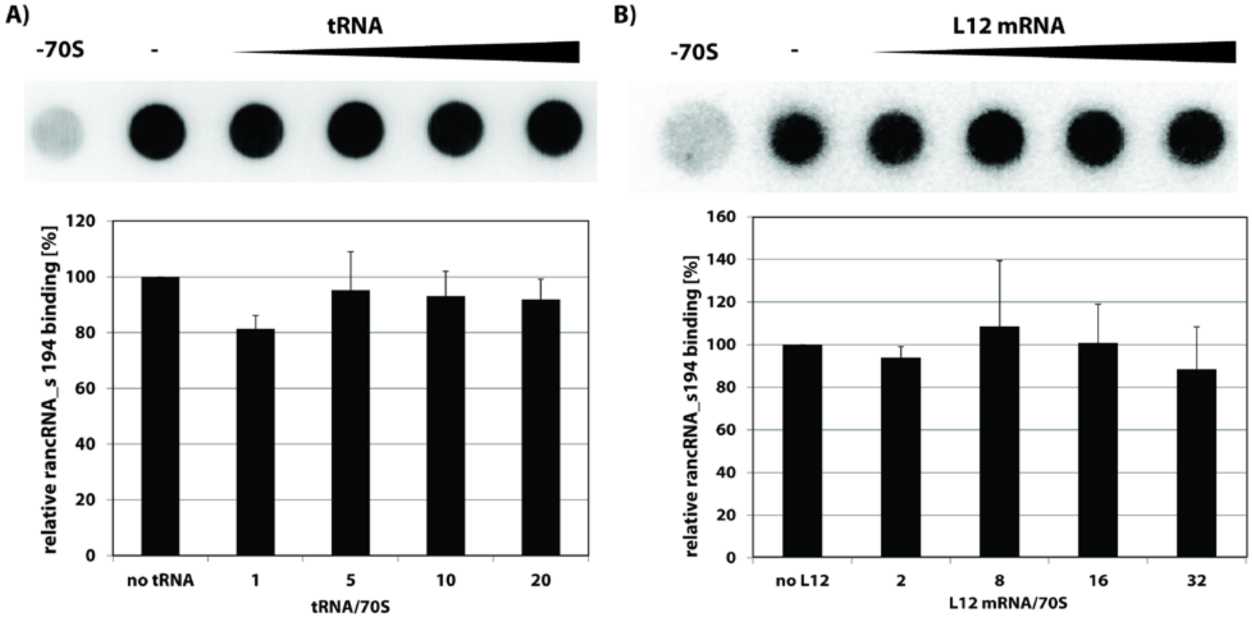

Supplementary Fig. 4. rancRNA_s194 does not compete with tRNA or mRNA for binding to the ribosome. **(A)** Radiolabeled rancRNA_s194 was bound to 5 pmol 70S ribosomes isolated from ΔrancRNA_s194 *H. volcanii* cells in the absence (-) or presence of increasing amounts of unlabeled yeast bulk tRNA. The mean and standard deviation of two independent filter binding experiments is shown. The added molar excess of tRNA over ribosomes is indicated. Signals measured in the absence of ribosomal particles (-70S) were subtracted from all experimental points. **(B)** Radiolabeled rancRNA_S194 binding to 5 pmol 70S ribosomes isolated from ΔrancRNA_s194 *H. volcanii* cells was measured in the absence (-) or presence of increasing amounts of unlabeled ribosomal protein L12 mRNA from *Methanococcus thermolithotrophicus*. The mean and standard deviation of two independent filter binding reactions experiments is shown. The added molar excess of mRNA over ribosomes is indicated.

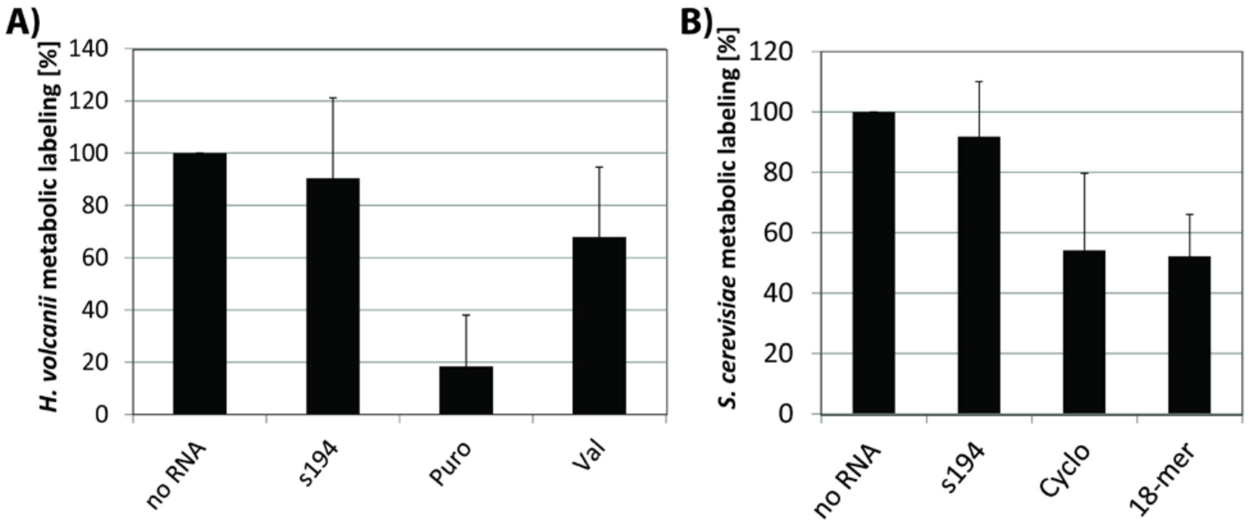

Supplementary Fig. 5. Metabolic labeling in the presence of rancRNA_s194 in *H. volcanii* (A) and *S. cerevisiae* (B). **(A)** Metabolic labeling shows that rancRNA_s194 does not inhibit *in vivo* translation in *H. volcanii*. Quantifications of four metabolic labeling experiments are shown using spheroplasts prepared from ΔrancRNA_s194 *H. volcanii* cells in the absence (no RNA) or in the presence of rancRNA_s194, or the Val-tRNA-derived fragment (Val) identified previously[2,3]. The antibiotic puromycin (Puro) served as translation inhibition control. **(B)** *H. volcanii* rancRNA_s194 was also unable to affect translation in yeast spheroplasts. In contrast, the *S. cerevisiae* mRNA-derived rancRNA_18 (18-mer)[4] inhibited translation to the same extent as the known translation inhibitor cycloheximide (Cyclo). The mean and standard deviations of four independent experiments are shown. Activities in the absence of any introduced synthetic RNA (no RNA) is set to 100%.

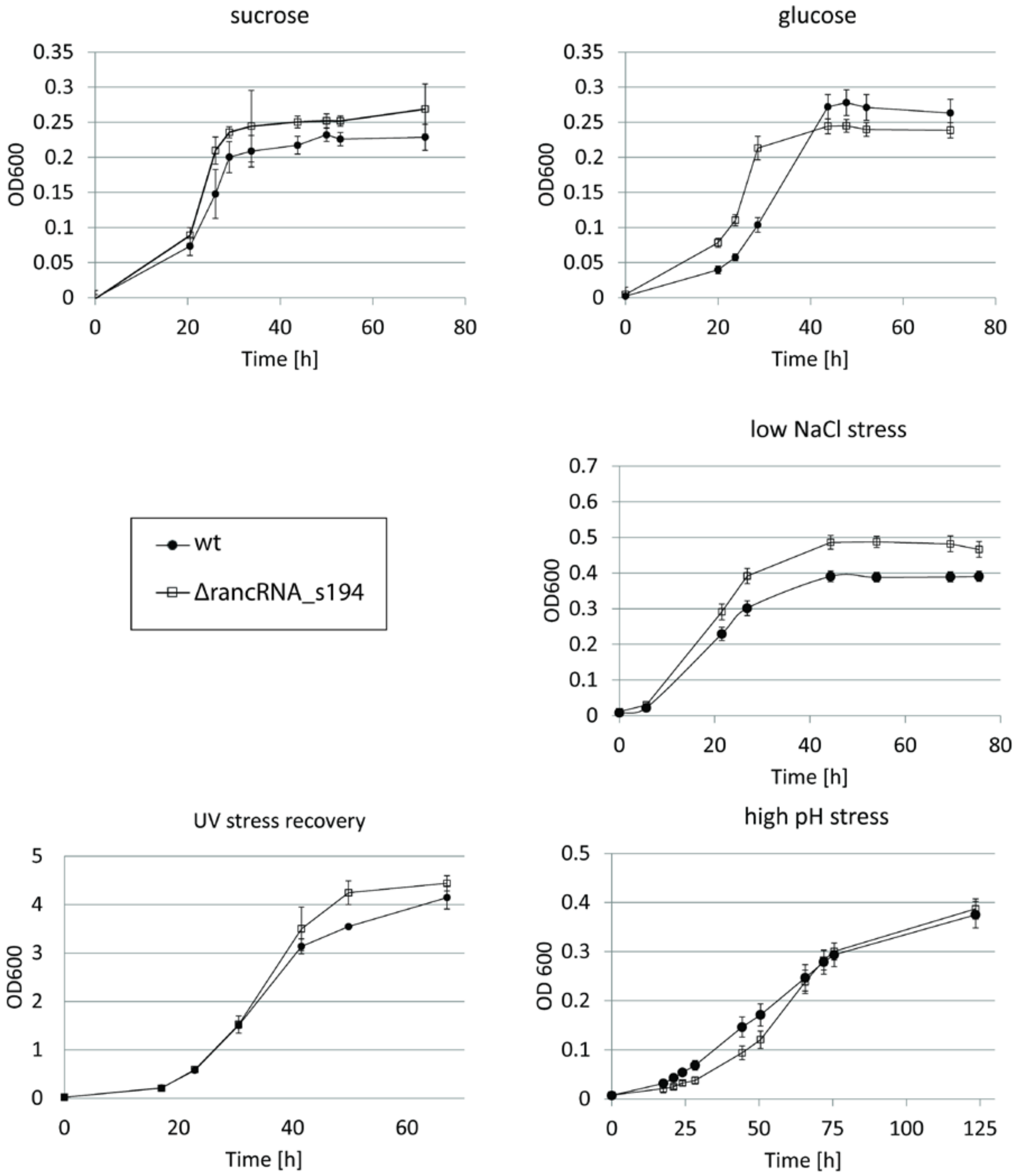

Supplementary Fig. 6. Growth curves of *H. volcanii* wildtype (wt) and rancRNA_s194 knock-out (ΔrancRNA_s194) cells were determined in synthetic medium containing sucrose or glucose as sole carbon source, or in full medium at low NaCl, alkaline pH, or after UV treatment. The mean and the standard deviations of three biological and seven technical replicates are shown.

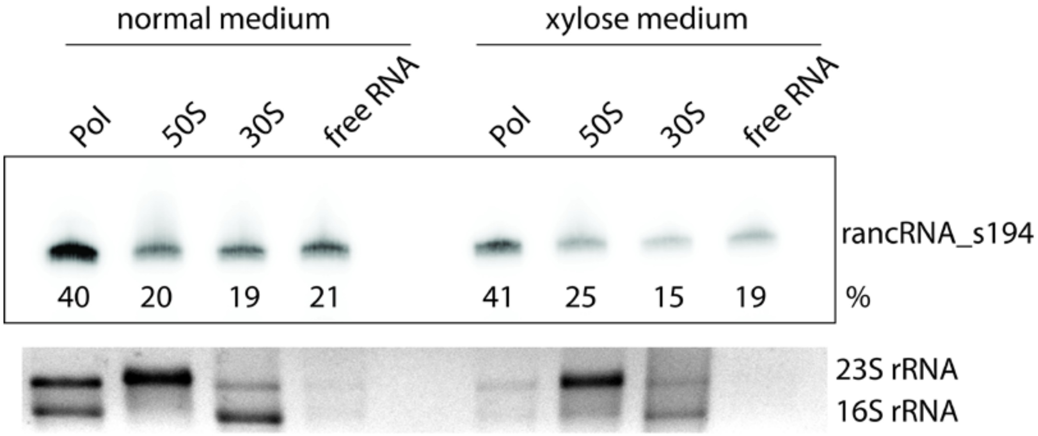

Supplementary Fig. 7. Association of rancRNA_s194 to ribosomal particles isolated from *H. volcanii* cells either grown in normal medium or synthetic medium containing xylose as sugar source. Northern blot (top) on RNA isolated from different sucrose gradient fractions containing polysomes (Pol), 50S or 30S ribosomal subunits, or light fractions (free RNA). Quantification of the northern blot signals is shown below the autoradiogram. Lower panel shows an agarose gel of ethidium bromide-stained 23S rRNA and 16S rRNA from each fraction. The rancRNA_s194 northern blot signal in the 30S subunit fraction most likely is due to a minor contamination of the small subunit preparation with 50S particles (see the faint 23S rRNA band in the 30S preparation on the agarose gel).

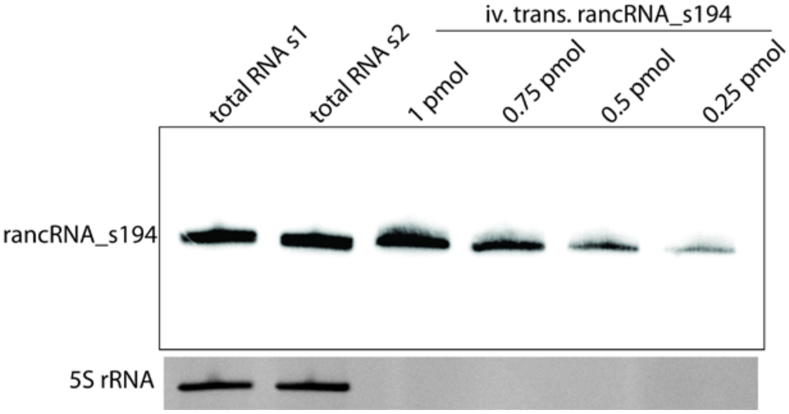

Supplementary Fig. 8. Estimation of the transcript number of rancRNA_s194 per *H. volcanii* cell. Total RNA was isolated from two biological replicates (s1/2) with known cell numbers (lanes 1-2 from the left). The 5S rRNA served as loading controls. Different amounts of *in vitro* transcribed rancRNA_s194 were loaded (lanes 3-6) and their northern blot signals were compared to the signal obtained from rancRNA_s194 in the total RNA samples. Thereby the rancRNA_s194 transcript number in the two biological replicates (lanes 1-2) could be calculated which was subsequently divided by the number of cells initially used.

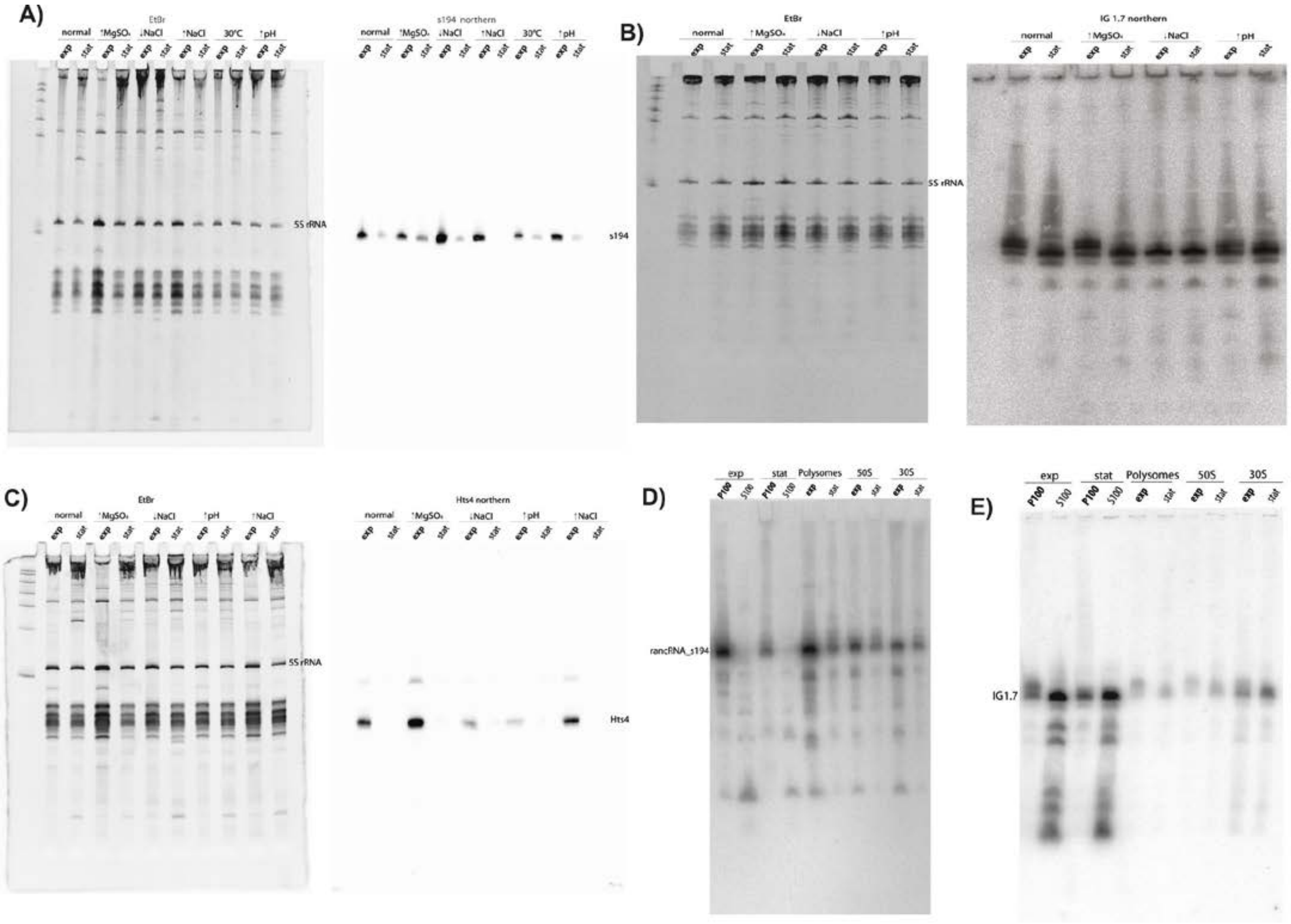
A)
EtBr
normal
↑MgSO4
↓NaCl
↑NaCl
30°C
↑pH
exp
stat
5S rRNA
s194 northern
s194
B)
IG 1.7 northern
IG1.7
C)
Hts4 northern
Hts4
D)
exp
stat
Polysomes
50S
30S
P100
S100
rancRNA_s194
E)
IG1.7

F)

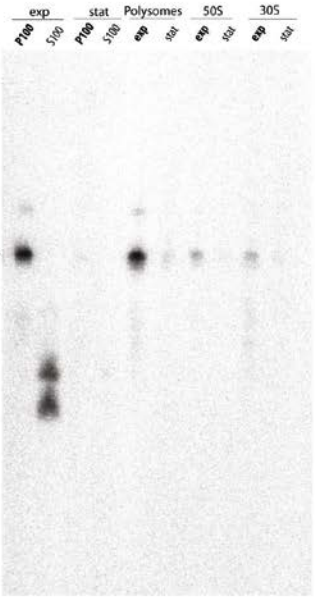

G)

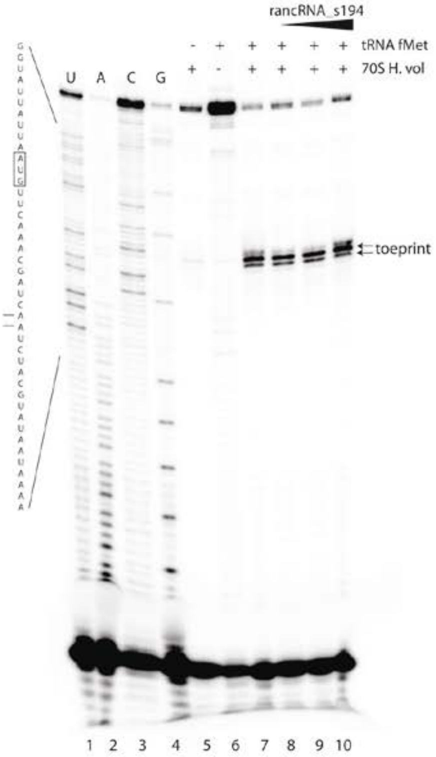

H)

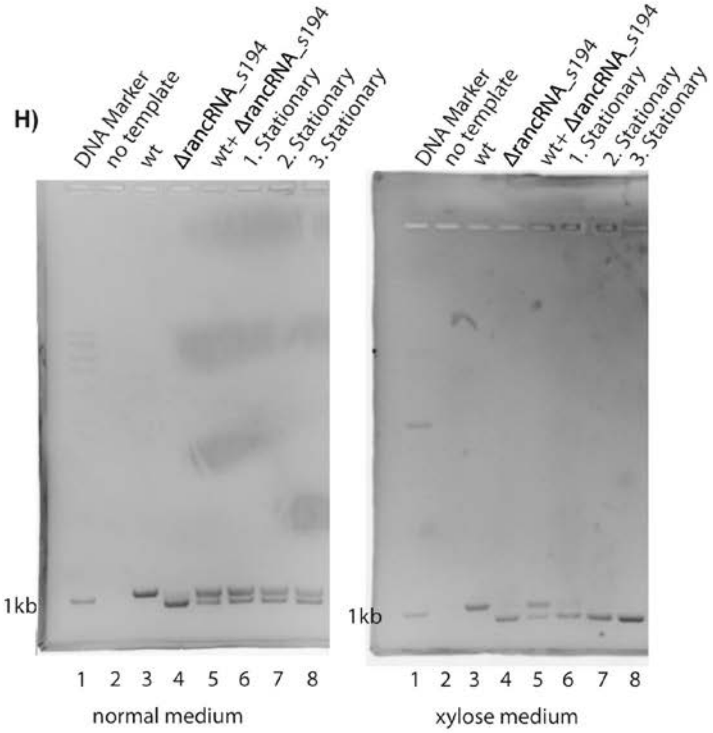

I)

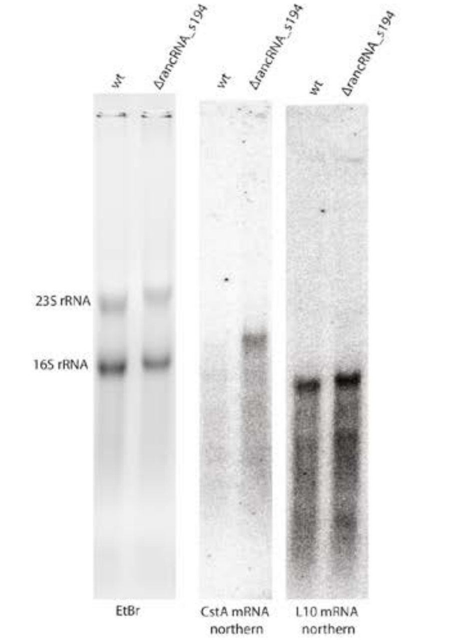

Supplementary Fig. 9. Uncropped and full-length gels and northern blots. The displayed gels and blot correspond to the following figures of the main text: **(A)** Figure 3A; **(B)** Figure 3B; **(C)** Figure 3C; **(D)** Figure 4A, B; **(E)** Figure 4A, B; **(F)** Figure 4A, B; **(G)** Figure 5B; **(H)** Figure 6B; **(I)** Figure 7C. For the corresponding details refer to the respective figure legends in the main text.

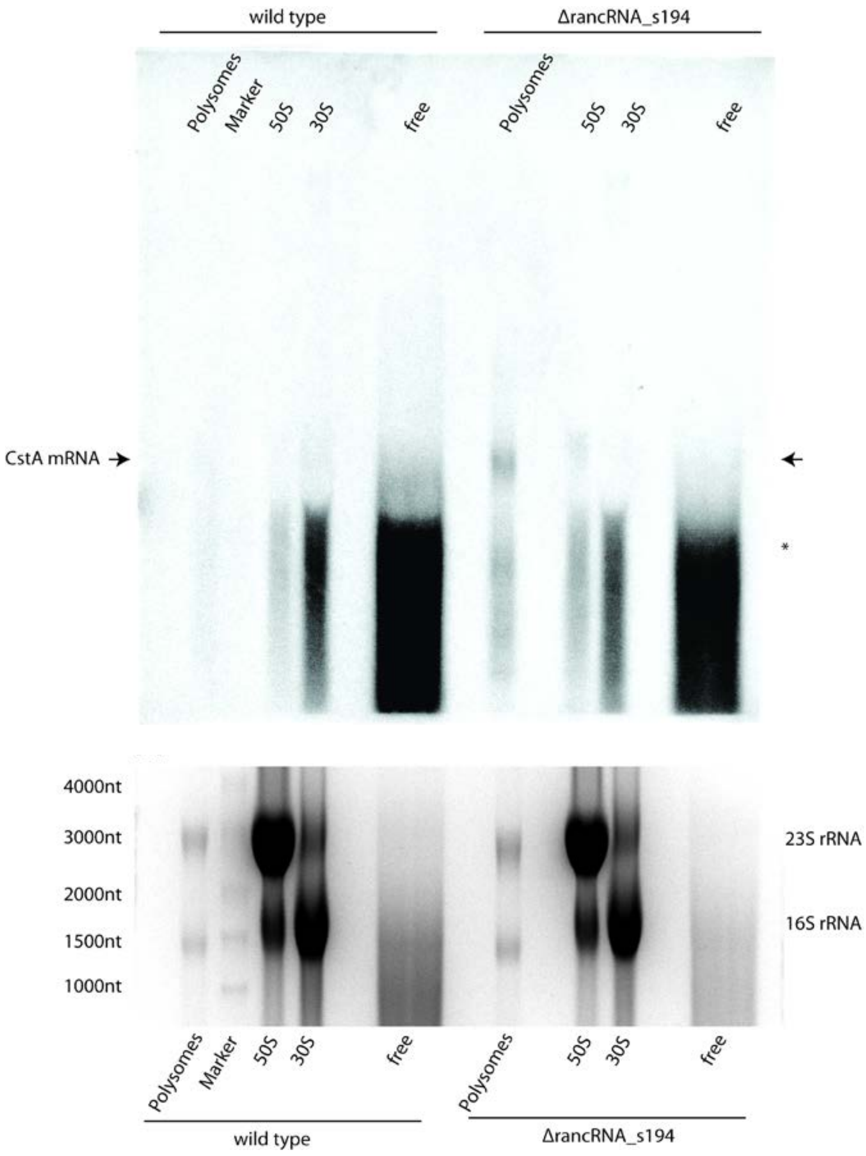

Supplementary Fig. 10. Uncropped and full-length northern blot. (top) The distribution of the *cstA* mRNA between different polysome gradient fractions was monitored by northern blot analysis and qualitatively compared between wildtype and rancRNA_s194 knock-out (Δ) *H. volcanii* cells. In all gradient fractions corresponding volumes were loaded, except for the light gradient fraction (free), where only 50% of the corresponding volume fraction was applied. The asterisk indicates a region of unspecific annealing of the northern blot probe. (bottom) The ethidium bromide-stained agarose gel before blotting to the nylon membrane shows 23 rRNA (2,915 nt) and 16S rRNA (1,473 nt) alongside an RNA size marker (Marker). The size of the *cstA* mRNA is 1,824 nt.

Supplementary Table 1: Used DNA oligonucleotides in this study

DNA Primer	Sequence 5'-3'
Northern Blot	
rancRNA_s194NB	TTACTCTGTACGGTGTTGCATCC
Hts4NB	CACCGAGGCCCAGCTATCCTGACC
IG1.7NB	TAACCTTCGTCATCGCCACC
mRNA northern	
CstA1NB	GCGTCTCGGGCGTCCCGACGATTTCCTCCATCATG
CstA2NB	GCGCAATCGGGAACTGAATCCCCGCGTAGACGCCG
to amplify part of the L10 gene	
L10fw	ACGTTGGAGTCTTCCGTCAC
L10rv	CAGTGGAAGCGTGAAGAGGT
to amplify part of HVO_2032 (ABC...)	
2032ABCfw	GTGTTGACGCCCCAAGAAGT
2032ABCrv	CCGACCCCTCGTACATGACT
in vitro transcription	
s194fw(T7)	GGATCCTAATACGACTCACTATAGGGATGCAACACCGTACAG
s194rv	TAAAGGGGTGACCGAACC
scr1T	GGATCCTAATACGACTCACTATAGGGGATGATAGATCTCTCGGCCATAGCAGTACGTTAGCACGCGCGAAAGCTAACAA
scr2T	CAGGCACACGACTGCAAGTCGCAGTCTTGCCTGTAGCATCAAGCTGAATAGTCCGCACCATTGTTAGCTTTCGCGCGTG
scr(rv)	TCGCAGTCTTGCCTGTAGCA
CstA_T7_fw	GGATCCTAATACGACTCACTATAGGGGCAGCGTACCATGG
CstA_T7_rv	AGACTCGCCGTGGCGGCC
Hvo_2032_T7fw	GGATCCTAATACGACTCACTATAATGACAGACACGACCGCAC
Hvo_2032_T7_rv	GACGGGACAGCTCGGTGATG
L10_T7_fw	GGATCCTAATACGACTCACTATAGGGATGAGCGAATCCGAGGTTCG
L10_T7_rv	ACGTTGGAGTCTTCCGTCAC
growth competition	
s194gc(fw)	CTCTTGACTCGACCCTCCTG
s194gc(rv)	AAGCGATACAGTCGCAGGTC
IG1.7_1n	GTCACCGACGACGGGAGAAT
IG1.7_4n	CGTCAACAAGGTCGGAAGCC
Knock out	
IG1.7P1n	GTCACCGACGACGGGAGAAT
IG1.7P2n	GGTGGATTCTTGAACGTCGTTCTATCGCTCCGAAT
IG1.7P3n	GAGCGATAGAACGACGTTCAAGAATCCACCCTTTT
IG1.7P4n	CGTCAACAAGGTCGGAAGCC

Material and Methods

Knock out of IG1.7

Deletion of the IG1.7 locus in *H. volcanii* H26 was generated using the ''pop-in/pop-out'' strategy as previously described[5,6]. In short, two fragments of around 250 nucleotides up and downstream of the intended deletion site on the main chromosome (chr: 1,781,263 - 1,781,362) were generated by PCR using genomic DNA as template. The following primer pairs were used: IG1.7P1n and IG1.7P2n, as well as IG1.7P3n and IG1.7P4n, respectively. Primers IG1.7P2n and IG1.7P3n had a 30 nucleotide long overlap to each other. In a second PCR reaction with primers IG1.7P1n and IG1.7P4n and the two first PCR products as template, a fusion fragment was generated. This fusion fragment was then cloned into plasmid pTA131 and used to transform *H. volcanii* H26 (see **Supplementary Table 1** for primer sequences).

Metabolic labeling To assess *in vivo* translation in *H. volcanii* in the absence or presence of rancRNA candidates, a metabolic labeling experiment was performed as described previously[2]. Metabolic labeling using S. cerevisiae spheroplasts was performed as described[4].

References

1 Jantzer, K., Zerulla, K. & Soppa, J. Phenotyping in the archaea: optimization of growth parameters and analysis of mutants of Haloferax volcanii. *FEMS Microbiol. Lett.* **322**, 123-130, (2011).

2 Gebetsberger, J., Wyss, L., Mleczko, A. M., Reuther, J. & Polacek, N. A tRNA-derived fragment competes with mRNA for ribosome binding and regulates translation during stress. *RNA Biol.* **14**, 1364-1373 (2017).

3 Gebetsberger, J., Zywicki, M., Kunzi, A. & Polacek, N. tRNA-derived fragments target the ribosome and function as regulatory non-coding RNA in Haloferax volcanii. *Archaea* **2012**, 260909 (2012).

4 Pircher, A., Bakowska-Zywicka, K., Schneider, L., Zywicki, M. & Polacek, N. An mRNA-Derived Noncoding RNA Targets and Regulates the Ribosome. *Mol Cell* **54**, 147-155 (2014).

5 Allers, T., Ngo, H. P., Mevarech, M. & Lloyd, R. G. Development of additional selectable markers for the halophilic archaeon Haloferax volcanii based on the leuB and trpA genes. *Appl. Environm. Microbiol.* **70**, 943-953 (2004).

6 Bitan-Banin, G., Ortenberg, R. & Mevarech, M. Development of a gene knockout system for the halophilic archaeon Haloferax volcanii by use of the pyrE gene. *J. Bacteriol.* **185**, 772-778 (2003).
